# Efficient recovery and DNA extraction for algae-associated microbial communities

**DOI:** 10.3389/fpls.2025.1693747

**Published:** 2026-01-05

**Authors:** Elizaveta Chevokina, Daria Sibiryakina, Andrey Sobolev, Darya Slonova, Alina Demkina, Daria Yurikova, Alina Galivondzhyan, Olga Konovalova, Dmitry Sutormin, Artem Isaev

**Affiliations:** 1 The Center for Bio- and Medical Technologies, Moscow, Russia; 2 Gamaleya National Research Centre of Epidemiology and Microbiology, Moscow, Russia; 3 Shemyakin-Ovchinnikov Institute of Bioorganic Chemistry, Moscow, Russia; 4 Marine Research Center of Lomonosov Moscow State University, Moscow, Russia; 5 Shirshov Institute of Oceanology, Russian Academy of Sciences, Moscow, Russia; 6 Faculty of Biology, Lomonosov Moscow State University, Moscow, Russia

**Keywords:** algae, biofilms, DNA extraction, metagenomics, microbial communities

## Abstract

The extraction of high-quality microbial DNA from environmental samples is critical for many downstream applications, including short- and long-read metagenomic sequencing. However, environmental DNA is prone to low recovery, degradation, and contamination by enzymatic inhibitors, with the extent of these issues largely dependent on the DNA purification method. The embedding of bacterial cells in a mucoid matrix within biofilms further complicates the process, making the study of algal symbionts particularly challenging. This study benchmarked five methods to recover microbial cells from biofilms associated with three major groups of marine macroalgae, namely: red (*Palmaria stenogona*), brown (*Saccharina japonica*), and green (*Ulva lactuca*). This was followed by a systematic evaluation of six widely used commercial DNA purification kits for their ability to extract high-quality DNA suitable for 16S rRNA gene and shotgun sequencing. A universal trade-off was observed between the quantity and quality of the extracted DNA. While whole-sample homogenization and manual collection of biofilms resulted in high levels of chloroplast contamination, washing microbial cells with a buffer led to low DNA recovery; however, the use of a detergent improved DNA yields. A comparison of the DNA extraction kits revealed that their efficiency varied significantly among algal species, with the GeneJET Genomic DNA Purification Kit (Thermo Scientific) identified as the most versatile. The present findings provide a comparative benchmark of methods to recover algae-associated microbial communities and extract their DNA, offering guidance in selecting procedures suited for metagenomic sequencing.

## Introduction

The global ocean represents the largest and most biodiverse ecosystem on Earth. Marine biodiversity remains poorly characterized, with significant knowledge gaps in regions such as the Arctic Ocean. Ice and water provide habitats for diverse organisms, including micro- and macroalgae. Marine algae and cyanobacteria form the base of the ocean food webs and are one of the major primary producers. Notably, ice-associated microalgae account for ~57% of primary production in the central Arctic Ocean (Gosselin et al., 1997; Lee et al., 2013). Macroalgae, also known as seaweed, are dominant in coastal ecosystems (Budge et al., 2008; Søreide et al., 2006) and create unique habitats that enhance local biodiversity. Marine algae also hold significant economic and biotechnological value. They are traditionally cultivated for food and as a source of biomaterials in the cosmetic industry. Bioactive compounds derived from seaweed, such as polysaccharides, pigments, and phenolic compounds, have been shown to possess antibiotic, antioxidant, and anti-cancer properties and may help manage diet-related metabolic conditions (Cornish and Garbary, 2010; Kasanah et al., 2015; Kim et al., 2019; Lins et al., 2009; Sørensen et al., 2019). Additionally, seaweeds are used as a promising source of livestock feed biomass, in biofuel production (Kumar et al., 2013), in bioremediation (Azeem et al., 2019), and as bioindicators (Corey et al., 2012).

This study focuses on three industrially and biotechnologically significant algal species, namely: the brown alga *Saccharina japonica* (Phaeophyceae), the green alga *Ulva lactuca* (Chlorophyceae), and the red alga *Palmaria stenogona* (Rhodophyceae). *S. japonica* is a valuable source of polysaccharides (laminarin, alginic acid, fucoidan), mannitol, and trace elements (Vishchuk et al., 2012) as well as a potential biofuel feedstock (Demirbas, 2010). It forms marine forests in Asian coastal ecosystems (Lindsey Zemke-White and Ohno, 1999) and is cultivated for medicinal applications due to its antiviral (Ponce et al., 2019), antioxidant (Mizuta and Yasui, 2010), anticancer (Anastyuk et al., 2017; Vishchuk et al., 2012), hypolipidemic (Ren et al., 2019), neuroprotective (Jin et al., 2013), and anti-inflammatory (Asanka Sanjeewa et al., 2019; Jayawardena et al., 2019) properties. *U. lactuca* produces bioactive compounds with antioxidant (Kazir et al., 2019), immunomodulatory (Cian et al., 2018), antiviral (Shi et al., 2017), anticancer (Kaeffer et al., 1999), and anticoagulant (Harada and Maeda, 1998; Mao et al., 2006) effects. Its structural polysaccharides (ulvans) have applications in pharmaceutics, biomedicine, and agriculture (Cian et al., 2018; Kazir et al., 2019). *Ulva* also shows promise as a biofuel source (Dominguez and Loret, 2019), given its high CO_2_ sequestration efficiency (Raikova et al., 2017) and oil content suitable for biodiesel (Soliman et al., 2018). Additionally, it accumulates heavy metals (e.g., Cu, Cr, Cd, Pb) (Ibrahim et al., 2016) and thrives in saltwater/wastewater (Dominguez and Loret, 2019). *Palmaria* species (e.g., *P. palmata*) are consumed as food (Mouritsen et al., 2013) and produce phycoerythrin used in cosmetics (Stévant et al., 2023). While they contain natural antioxidants (Yuan et al., 2005), their antioxidant capacity is lower than that of brown algae (Roleda et al., 2019).

Algae host diverse microbial communities, which vary in composition depending on the algal species, environmental conditions, and the microhabitats on their surfaces. Despite this variability, macroalgae and bacteria exhibit tight ecological linkages (Bengtsson et al., 2012). Notably, bacterial communities associated with algae of the same species show conserved patterns across geographically distinct populations (Lachnit et al., 2009). Microbial communities could provide their algal host with nutrients and fixed nitrogen and can even communicate with the host using chemical signaling (Lachnit et al., 2009; Wu et al., 2016). Some bacterial species could be essential for algae development, and disbalance in algal microbiota could lead to disease (Gachon et al., 2010). The composition of algal microbiota is hypothesized to be regulated by secondary metabolites produced by both host macroalgae and associated bacteria (Bengtsson et al., 2012). Microbial communities on the algal surface typically form structured biofilms embedded within a complex extracellular matrix composed of exopolysaccharides and other polymeric substances (Aminina et al., 2020; Liu et al., 2016; Seymour et al., 2017; Xia et al., 2020). This matrix provides microbial cells with protection against environmental stressors and contributes to biofilm stability. A complex network of interactions between bacteria, algal hosts, and microalgae commensals supports the conceptualization of these systems as holobionts (Egan et al., 2013). However, the highly organized and protected nature of these biofilms presents significant technical challenges for their study.

The selection of an appropriate DNA extraction method represents a critical step in metagenomic studies, as different sample types often require specialized protocols. DNA quality parameters—including purity, fragment size, and the absence of contaminants and enzymatic inhibitors—significantly influence downstream applications such as long-read sequencing (Demkina et al., 2023; Pearman et al., 2024). Notably, all DNA extraction methods introduce inherent biases in microbial community representation and may contribute to kit-specific contaminants (Demkina et al., 2023). These challenges are particularly pronounced when sequencing algae-associated biofilms for the following reasons: (i) microbial cell extraction is hindered by strong adhesion to algal surfaces and matrix entrapment, (ii) contamination with host DNA diminishes the quality of sequencing data, (iii) a specific problem for 16S metagenomic sequencing is the similarity between microbial and chloroplast rRNA gene sequences that results in the amplification of both microbial and host DNA with standard primers (Hanshew et al., 2013; Regalado et al., 2020; Thomas et al., 2020), and (iv) sequencing inhibition occurs due to co-extracted matrix components (e.g., polyphenolic compounds) (Pearman et al., 2024; Schrader et al., 2012). These challenges of DNA extraction from algal and other types of microbial biofilms are discussed in the literature, highlighting the need for the development of an optimized DNA purification procedure (Burke et al., 2009; Corcoll et al., 2017; Govil et al., 2019).

**Figure 1:**
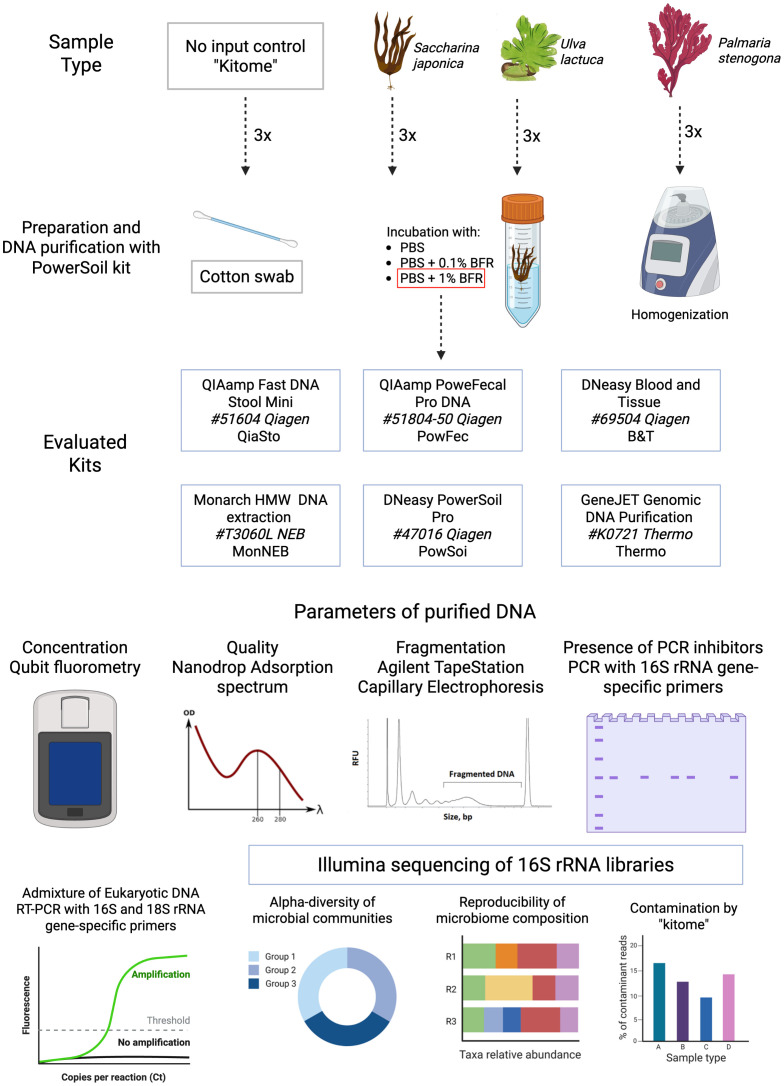
A schematic representation of the methodology used in the study. Samples from three species of macroalgae (*S. japonica*, *U. lactuca*, and *P. stenogona*) were collected and processed with the PowerSoil kit in three technical replicates using five types of sample preparation methods (washing with different concentrations of detergent, swabbing, and whole-sample homogenization). Downstream analysis was performed with PBS + 1% BFR treatment. DNA from the same three sample types was extracted in triplicate with six commercially available kits. In addition, a no-input negative control was included to estimate kit-specific contamination. All samples were evaluated for an indicated set of parameters to select the best DNA purification strategy.

To address this gap, this study develops a standardized protocol for DNA extraction from microbial biofilms associated with three macroalgal species representing distinct phylogenetic groups (**Figure 1**). First, three microbial community recovery approaches were evaluated, namely: (1) swab extraction, which is labor-intensive yet is supposed to minimize host cell contamination, (2) whole-sample homogenization, which maximizes yield but introduces substantial host DNA contamination, and (3) detergent-based treatment followed by microbial cell collection, which was identified as the most balanced method. Using this optimized recovery method, six commercially available DNA extraction kits were evaluated for their performance with algal biofilm samples. Multiple criteria of extracted DNA were assessed (quantity, fragmentation, presence of PCR inhibitors, and admixture of chloroplast DNA), and the resulting samples were subjected to 16S rRNA sequencing to evaluate kit-introduced biases in microbial community composition. These results should guide the selection of appropriate DNA extraction methods for the studies of epiphytic microbial communities of marine macroalgae.

## Materials and methods

### Collection of samples

#### Macroalgae samples

All macroalgae samples were collected in June–July 2023 at the littoral zone of the Sea of Okhotsk (**Table 1**, **Supplementary Figure S1**). The samples were individually packed in sterile 50-mL falcon tubes, and up to five replicates were collected from the same thallus, transferred, and stored at -20°C until further processing. Although specific storage solutions were not applied, fresh-frozen algal material stored at low temperature is often considered sufficient for microbial DNA extraction (Burgunter-Delamare et al., 2022). These samples were subjected to five bacterial cell recovery methods and six commercially available DNA extraction kits. In addition, no-input negative controls (“kitomes”) were included to estimate the potential contamination of a microbiome sample during processing (**Figure 1**).

**Table 1:** Metadata of macroalgae samples: macroalgae species, collection site location, and collection dates.

Macroalgae species	Location	Collection site coordinates	Sample collection date
*Saccharina japonica*	Sea of Okhotsk, Magadan	59.1641° N, 151.6501° E	28.06.2023
*Ulva lactuca*		59.1173° N, 151.3526° E	29.06.2023
*Palmaria stenogona*		58.9865° N, 151.2428° E	02.07.2023

#### “Kitomes”

For any bacterial cell recovery condition and any DNA extraction kit tested, a “kitome” control was prepared in a single replicate. For this, no starting material was added except for the corresponding buffers (PBS, BFR, and lysis buffers) for DNA extraction. The data about the composition of each “kitome” was further used to evaluate the contamination introduced by each kit and to eliminate non-related taxa in a downstream analysis.

### Recovery of bacterial cells from macroalgae material

Samples collected in the field were kept at -20 °C for the duration of the transfer (up to 3 months). After thawing, rhizoids and zones of growth were removed, and the macroalgae thallus was cut into similar-sized fragments. Approximately 1.5 to 5 g of material has been obtained from each sample for downstream processing (**Supplementary Tables S1, S2**). Five methods were used to recover bacterial cells from the macroalgae thalli. Three replicates from the same thallus were used for each purification procedure.

Incubation in a buffer: The macroalgae material was perfused with sterile phosphate-buffered saline (PBS) or PBS containing 0.1% or 1% BFR and incubated for 2 h at 25 °C with shaking at 180 rpm. A low concentration of the BFR reagent was used to avoid extensive damaging of bacterial cells. The BFR reagent (BFR PEROXYVIR, produced by “BFR Labs”, Russia) is a mixture of enzymes (lyase and esterase), detergents, and oxygen-proactive compounds advertised to be efficient at biofilm removal (Emshanov et al., 2022). The BFR reagent can be substituted with other types of disinfectant/detergent treatments. The sample was then centrifuged at 300 g for 15 min to remove large debris and macroalgae fragments. The supernatant was collected and centrifuged again at 10,000 *g* for 10 min at 4 °C to pellet bacteria.

Swab method: The macroalgae material was wiped with a sterile medical swab, and the swab was washed in Qiagen PowerSoil Pro CD1 buffer.

Homogenization method: The macroalgae material (the sample weight is indicated in **Supplementary Table S1**) was homogenized using Tissue Lyzer (Bertine Technologies) for 4 min at 4,500 rpm for four cycles, with each cycle consisting of 30 s of homogenization followed by a 30-s pause.

### Confocal microscopy

Fragments of macroalgae thallus before and after recovery of bacterial cells were stained in 5 μM SYTO9 nucleic acid stain (Invitrogen) according to the manufacturer’s instructions. The fragments were immobilized on agarose pads (1.2% agarose in PBS) and imaged at ×100 magnification using a Nikon Eclipse Ti microscope equipped with the Nikon Plan Apo VC 100 × 1.40 oil objective and Nikon DS-Qi2 digital monochrome camera. The images were processed using ImageJ2 v2.35 software (Schneider et al., 2012).

### DNA extraction

All DNA extraction steps were carried out in accordance with the manufacturer’s instructions. Specific minor modifications to the standard protocol are mentioned. The sample weights used are indicated in **Supplementary Tables S1, S2**. For all stages requiring water, sterile nuclease-free water (B1500L, NEB) was used.

QIAamp Fast DNA Stool Mini Kit (#51604, Qiagen), further referred to as QiaSto kit: The isolation of DNA begins with the removal of PCR inhibitors and other chemicals that may interfere with subsequent enzymatic reactions. Following this, bacterial cells are lysed, and proteins are removed via proteinase K treatment. The DNA is transferred to the QIAamp spin silica column, washed twice, and eluted with a buffer. This kit was selected for the study due to its performance in previous observations (Demkina et al., 2023) and the PCR inhibitors’ removal step in the protocol.

QIAamp PowerFecal Pro DNA kit (#51804-50, Qiagen), further referred to as PowFec kit: At the first stage, cells are disrupted both mechanically (bead beating) and chemically (using lysis buffer). Next, PCR inhibitors are removed from the sample using Inhibitor Removal Technology^®^ in order to prevent interference with downstream enzymatic reactions. Then, DNA is isolated from the supernatant using the MB spin silica column, and the silica membrane is washed twice before elution. This kit was selected for the study due to its frequent consideration as “gold standard” for a diverse set of samples (Child et al., 2024; Demkina et al., 2023; Shaffer et al., 2022) and the presence of PCR inhibitors’ removal step in the protocol.

DNeasy PowerSoil Pro Kit (#47016, Qiagen), further referred to as PowSoi kit: The protocol begins with mechanical (bead beating) and chemical (lysis buffer) disruption of cells. The kit also includes a PCR inhibitor-removing step involving Inhibitor Removal Technology^®^. DNA is then captured from the supernatant using the MB spin silica column. After two rounds of washing, the DNA is eluted from the silica membrane. This kit was selected for the study due to its frequent consideration as “gold standard” for a diverse set of samples (Child et al., 2024; Demkina et al., 2023; Shaffer et al., 2022) and the presence of PCR inhibitors’ removal step in the protocol.

DNeasy Blood and Tissue kit (#69504, Qiagen), further referred to as B&T kit: After cell lysis using the STET buffer (50 mM Tris-HCl, pH 8.0; 50 mM EDTA, pH 8.0; 5% Triton X-100; 200 mM NaCl) supplemented with lysozyme (10 mg/mL), the sample is loaded onto a DNeasy Mini spin silica column. The DNA is washed twice and then eluted from the silica membrane. This kit was selected for the study due to its performance in previous observations (Demkina et al., 2023) and the necessity to test the performance of different DNA extraction kits produced by the same manufacturer (Qiagen).

Monarch HMW DNA extraction kit for Tissue (#T3060L, NEB), further referred to as MonNEB kit: The homogenization step of the protocol was omitted, and DNA isolation was performed using the recommended protocol for bacterial cells. All samples were incubated in the STET buffer with lysozyme (10 mg/mL) and heat-treated according to the manufacturer’s recommendations. Next, the lysis master mix solution was added to the samples, followed by the removal of proteins and RNA. DNA was extracted using the glass beads provided with the kit. Finally, the purified DNA was gently washed by rotation and eluted from the beads. This kit was selected because it is designed for the extraction of high-molecular-weight DNA, typically yielding higher DNA integrity numbers (DIN) and making it suitable for evaluating potential long-read sequencing applications.

GeneJET Genomic DNA Purification Kit (#K0721, ThermoFisher Scientific), further referred to as Thermo kit: To isolate DNA using this kit, a version of a protocol recommended for gram-negative bacteria was followed. Bacterial cells were lysed using a chemical method, as recommended by the manufacturer. The prepared lysate was then transferred to a GeneJET genomic DNA purification silica column. The column was washed twice, and the purified DNA was eluted from the silica membrane. The selection of this kit for the study was driven by its protocol simplicity and the cost per sample (**Supplementary Table S3**), providing a cost-effective and easily applied alternative for other DNA extraction kits.

For all kits, DNA was eluted in a 60-μL elution buffer supplied in a corresponding kit, and the eluate was then re-applied to a silica column or beads to repeat the elution step in order to maximize the DNA yield.

The selection of DNA extraction kits was mostly driven by their wide application in metagenomics and the specific protocol steps.

### DNA quantification and quality estimation

DNA concentration was measured using the Qubit 4.0 fluorometer (Invitrogen) with the Qubit dsDNA High Sensitivity Assay Kit or Qubit dsDNA Broad Range Assay Kit. If DNA concentration was below the detection limit for the Qubit dsDNA High Sensitivity Assay Kit, it was set to 0.1 ng/µL. For DNA yields per gram of input material, median and interquartile range (IQR) were calculated due to high variance. DNA purity was determined by measuring the ratios of absorbance at 260 and 230 nm (260/230) and at 260 and 280 nm (260/280) using the NanoDrop 1000 spectrophotometer. Samples were considered pure if they had the 260/230 ratio between 2.0 and 2.2 and the 260/280 ratio between 1.7 and 2.0 (Demkina et al., 2023).

The integrity of DNA has been assessed using the Agilent TapeStation 4150 (Agilent Technologies) and the Genomic DNA ScreenTape system, following the manufacturer’s instructions. Furthermore, 1 µL of a sample with a concentration >7 ng/μL was used directly for the assay. Samples with DNA concentration <7 ng/μL were concentrated using the HyperVAC concentrator beforehand. Samples with a DIN value above 7.0 could be considered as “high quality” and suitable for long-read sequencing.

### PCR assay for the detection of PCR inhibitors

The V3–V4 region of the 16S rRNA gene was PCR amplified using universal Illumina V3/V4 primers (**Supplementary Table S3**). Each PCR reaction contained 1 μL of DNA (or DNA diluted 10–100 times with NEB nuclease-free water), 0.25 μL of forward and reverse primer (final concentration of 0.5 mM), 5 μL of Phusion High-Fidelity PCR Master Mix with HF Buffer (NEB), and 3.5 μL of nuclease-free water (NEB) for a final reaction volume of 10 μL. The PCR cycling conditions were as follows: 98 °C for 30 s, 25 cycles of 98 °C for 10 s, 55 °C for 30 s, and 72 °C for 30 s, with a final 5-min elongation step at 72 °C. The PCR products were visualized using agarose gel (1.5% agarose in 1× Tris-EDTA buffer) electrophoresis and stained with ethidium bromide (0.25 μg/mL).

### Preparation of 16S rRNA libraries, sequencing, and data analysis

Amplification of the V3–V4 region of 16S rRNA and library preparations were performed according to the Illumina manual (Illumina, 2013). The amplicon libraries were barcoded, pooled, and sequenced using NovaSeq 6000, 2 × 250-bp paired-end protocol at Evrogen JCS.

For 16S data analysis, a snakemake pipeline for paired-end 16S sequencing data processing was developed (https://github.com/chorzow/16S_PE/) (Demkina et al., 2023). Briefly, the quality of sequencing data was estimated using FastQC, and then reads were trimmed and filtered using Trimmomatic v0.39 (Bolger et al., 2014) in paired-end mode with the following parameters: -phred 33 ILLUMINACLIP:2:30:10 SLIDINGWINDOW:4:15 HEADCROP:17 MINLEN:150. Forward and reverse reads that passed quality control were further processed with the DADA2 pipeline v1.26.0 (Callahan et al., 2016). The resultant denoised, merged, and non-chimeric amplicon sequence variants (ASVs) were clustered using MMseqs2 v13.45111 (Steinegger and Söding, 2017) (coverage >0.95, identity >0.98), and representative sequences were further treated as operational taxonomic units (OTUs). OTUs were returned to DADA2, and taxonomy was assigned to OTUs using SILVA SSU database v.138 (Demkina et al., 2023; Quast et al., 2012). Contamination was removed using R package decontam v. 1.13.0 (Davis et al., 2018) in the “either” mode with a threshold of 0.3. Chloroplast sequences were removed by subtracting OTUs whose taxonomy on the order level was assigned to ‘Chloroplast’. Principal coordinate analysis (PCoA), alpha-diversity and taxonomic analyses were performed with R packages config v0.3.2, here v1.0.1, dplyr v1.1.4, ggplot2 v3.5.0, ggsci v3.2.0, lemon v0.4.9, patchwork v1.2.0, microshades v1.13, phyloseq v1.46.0 (McMurdie and Holmes, 2013), scales v1.3.0, jsonlite v2.0.0, reshape2 v1.4.4, cowplot v1.1.3, and vegan v2.6.2. Shannon index, calculated with phyloseq, was used as a metric that incorporates the richness and dominance of OTUs in a community. Reproducibility analysis was performed using Python v3.11. The statistical tests used are provided in brackets together with the reported *p*-values.

### Ranking of kits

To rank the DNA extraction kits, several approaches were employed. For DNA concentration, DNA integrity number (DIN), and Shannon index, ranking was based on the mean values obtained for the parameters measured, with rank 1 assigned to the sample exhibiting the highest mean value. For ranking of contamination levels, rank 1 (the top rank) was assigned for kits that exhibited contamination levels below the 1% threshold for all three replicates. The remaining kits were ranked based on their mean contamination levels, with lower ranks assigned to the sample with lower contamination levels. To rank reproducibility levels, the range between the highest and lowest reproducibility levels was divided into six equal bins for each macroalgae species independently. Then, kit ranks were assigned so that rank 1 was attributed to a kit that produced samples falling into a bin with the highest reproducibility levels.

## Results

### Microbial cells recovery from algae-associated biofilms

During a large-scale analysis of marine samples from the Arctic and Pacific oceans conducted by our laboratory, it was noticed that algae-associated microbial communities, extracted via incubation of algae with the PBS buffer, produced the lowest DNA yields and poor-quality 16S rRNA sequencing libraries. It was hypothesized that the reason could be the stronger attachment of microbial biofilms to the algal hosts and the presence of PCR inhibitors among the biofilm matrix compounds. To identify the most efficient method for obtaining high-quality metagenomic bacterial DNA from macroalgae thalli, we focused on samples from three different macroalgae species: *U. lactuca*, *S. japonica*, and *P. stenogona*. Algae material was collected at the shores of the Sea of Okhotsk during the summer of 2023 (**Table 1**, **Supplementary Figure S1**) and, before processing, was stored at -20 °C for 3 months.

First, different approaches to the recovery of microbial cells were compared (**Figure 1**): (1) collection of microbial cells with a sterile swab, which was expected to minimize host DNA contamination, (2) whole-sample homogenization, which was expected to maximize DNA yield but will introduce substantial host DNA contamination, and (3) detachment of bacterial cells after incubation of an algae thallus with a PBS buffer followed by cell collection via centrifugation. To enhance bacterial cell recovery from biofilms with a third method, a treatment with BFR reagent was used. This solution contains enzymes (lyase and esterase), detergents, and oxygen-proactive compounds expected to disrupt the biofilm matrix (Emshanov et al., 2022).

Initially, the efficiency of microbial cells’ removal from algae thalli was evaluated using confocal microscopy with DNA staining SYTO9 dye. Due to the high fluorescence of the host cells, bacteria were efficiently located only on the surface of *S. japonica* samples (**Supplementary Figure S2**). The number of microbial cells before and after treatment was compared, and incubation with PBS+BFR efficiently reduced visible titer, while incubation with PBS alone had minuscule effects. In addition, the swabbing resulted in damaging of the host cells that could result in increased eukaryotic DNA contamination.

The samples after each type of treatment were taken for 16S amplicon sequencing. The microbial DNA from algae samples was extracted using the PowerSoil kit (Qiagen), which was previously identified as one of the best-performing kits for complex types of environmental samples (Demkina et al., 2023).

#### DNA yield and quality

**Figure 2:**
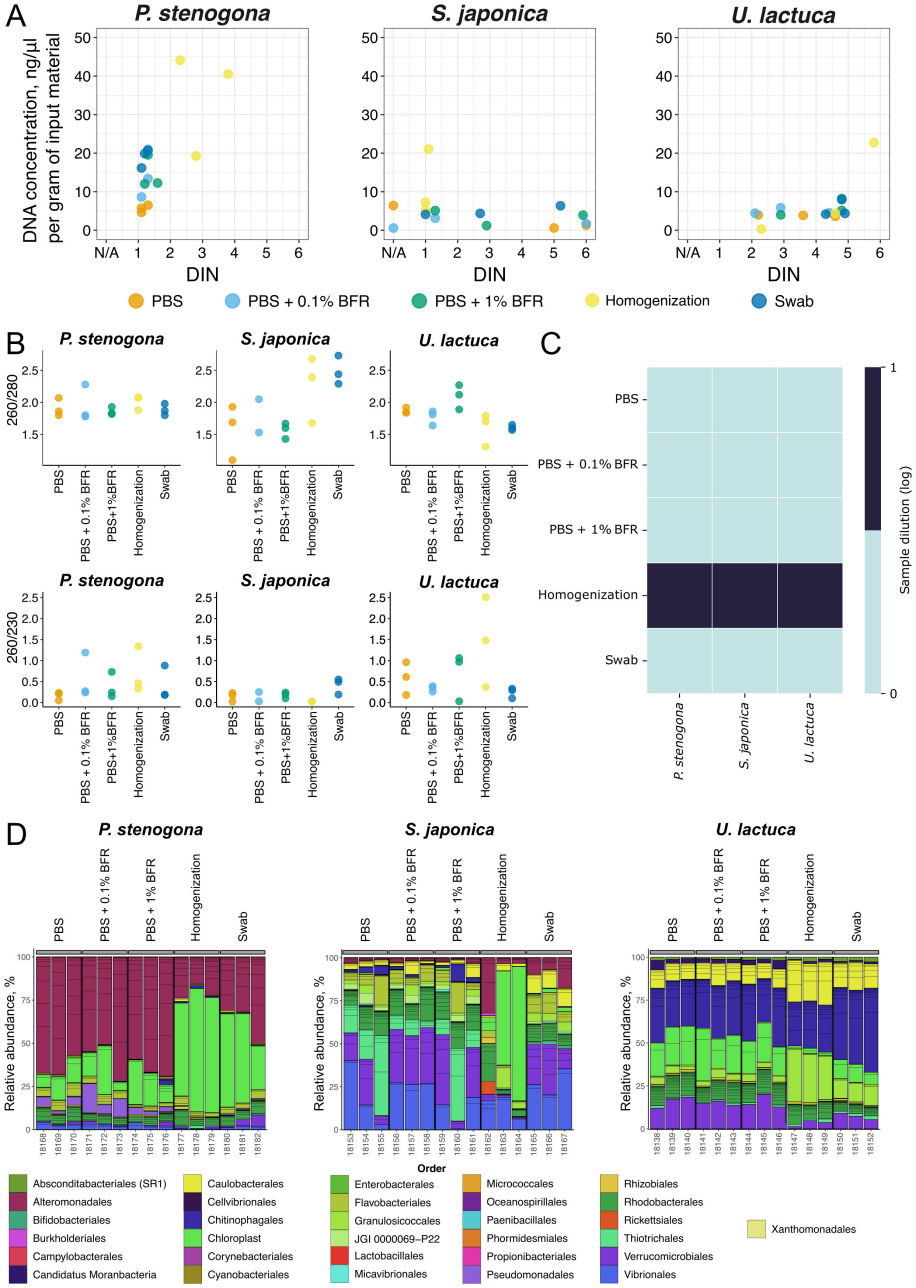
Benchmark of bacterial cell recovery methods. **(A)** DNA concentration and DNA integrity number (DIN) values for samples obtained using different recovery methods. Data for three technical replicates are shown. N/A on the x-axis indicates samples where DIN could not be measured on TapeStation due to low DNA quality. **(B)** DNA purity assessed by 260/280 (top) and 260/230 (bottom) absorption ratios. Data for three technical replicates are shown. **(C)** Presence of PCR inhibitors, estimated as a dilution factor required to achieve visible PCR amplification of the 16S rRNA gene region. An average of three technical replicates is shown. **(D)** Relative abundance of bacterial orders in the macroalgae samples. Bacterial orders with relative abundances >1% are shown. Data for three technical replicates are shown.

On average, algae samples generated relatively low yields of DNA: the mean yield for *P. stenogona* was 350.4 ng per gram of input material (SEM 77.8), 289.3 ng per gram of input material (SEM 77.5) for *S. japonica*, and 1,057.8 ng per gram of input material (SEM 178.2) for *U. lactuca* (**Figure 2A**, **Supplementary Table S1**). DNA integrity was very low for *P. stenogona* samples (DIN <2 for most of the treatments). *S. japonica* and *U. lactuca* samples demonstrated better values (mean DIN = 2.69 and 3.93, respectively) (*p* < 0.05, ANOVA); however, estimation of DINs at low DNA concentration could be compromised (**Figure 2A**, **Supplementary Table S1**, **Supplementary Figure S3**). The only obvious difference between treatment types was noticed for the homogenization method with *P. stenogona* samples, which generated higher DNA yields (*p* < 0.05, ANOVA) that still could be associated with increased contamination with the host DNA. Incubation with PBS was the least efficient method, whereas incubation with PBS + 0.1% BFR increased the mean DNA recovery by 155%, and 1% BFR further improved DNA yields by 2% compared to 0.1% BFR (**Figure 2A**).

Judging by the 260/280 ratio, *P. stenogona* samples recovered better-quality DNA with low protein contamination, while for the two other species this parameter was more dispersed (*p* < 0.01, Levene’s test), depending on the treatment type (**Supplementary Table S1**, **Figure 2B**). At the same time, the 260/230 ratio was substantially low for most of the treatment types, indicating a significant contamination with organic compounds (**Supplementary Table S1**, **Figure 2B**). To determine if the presence of impurities could affect downstream enzymatic reactions, 16S rRNA PCR with undiluted and diluted DNA samples was performed. This method could represent a rough proxy for the estimation of contamination since some types of environmental samples could be amplified only after 100× dilution (Demkina et al., 2023). Nevertheless, for all tested macroalgae species and for all types of treatment, except homogenization, undiluted samples were sufficient for the amplification (**Figure 2C**, **Supplementary Figure S4**). Samples after homogenization were amplified only after 10× dilution, indicating an admixture of PCR-inhibiting compounds.

Sequencing of the V3–V4 region of the bacterial 16S rRNA gene is a universal approach for the determination of microbial community composition (Ramazzotti and Bacci, 2018). For the plant- and algae-associated samples, this approach could also result in the amplification of chloroplast DNA (Hanshew et al., 2013). Chloroplast-excluding primers have been proposed (Artimová et al., 2022); however, considering that V3–V4 16S sequencing allows a direct comparison between different sample types (e.g., water from the environment and non-algae associated communities), it was decided to use standard V3–V4 primers in this study.

All samples were subjected to 16S amplicon sequencing, and the level of eukaryotic DNA contamination was estimated by measuring the proportion of reads mapping to chloroplast DNA (**Figure 2D**). For *P. stenogona* samples, a clear difference between treatments was observed (*p* < 0.01, ANOVA). Samples obtained with homogenization and swabbing contained a drastically increased amount of chloroplast DNA compared with three PBS-based sample preparation methods (*p* < 0.05, *T*-test) (**Figure 2D**). For *S. japonica*, two of the three replicates prepared with homogenization exhibited the same behavior, having more chloroplast DNA in their composition than all other preparation methods (*p* < 0.05, ANOVA). Swabbing samples demonstrated more chloroplast reads compared to PBS-based methods (*p* < 0.05, ANOVA), although not as abundant as in the homogenization samples (**Figure 2D**). Contrary to expectations, *U. lactuca* samples showed a different pattern. Here homogenization did not result in a significant overrepresentation of the chloroplast DNA.

An analysis of the microbial community’s composition revealed that PBS-based methods had consistently recovered similar taxonomic groups, whereas homogenization and swabbing demonstrated increased relative percentage of Granulosicoccales for *P. stenogona* and *U. lactuca* samples (*p* < 0.05, Mann–Whitney *U*-test) and decreased relative amounts of Thiotrichales for *S. japonica* (*p* < 0.05, Mann–Whitney *U*-test) and Verrucomicrobiales for *U. lactuca* samples (*p* < 0.05, Mann–Whitney *U*-test) (**Figure 2D**). Based on the sum of data (microscopy, DNA yields, the lack of DNA contamination with PCR inhibitors, and reduced quantities of chloroplast DNA), the treatment with PBS + 1% BFR produced DNA of sufficient quality while avoiding extensive host DNA contamination associated with the whole-sample homogenization approach. Therefore, this method was selected for the downstream analysis. However, for *U. lactuca* and especially for *S. japonica* samples, DNA yields were quite low, prompting further investigation into whether alternative DNA purification kits could improve the quantity and quality of DNA recovered after treatment of macroalgal thalli with PBS + 1% BFR (**Figure 1**).

### Benchmarking of DNA purification kits

After selecting the cell recovery method (incubation of macroalgae thallus in the PBS supplemented with 1% BFR followed by microbial fraction collection via centrifugation), six widely applied DNA purification kits were compared to identify the most suitable procedure for the extraction of metagenomic DNA. Five of these kits utilize spin columns for DNA extraction and purification (PowSoi, PowFec, QiaSto, B&T, Thermo), while the MonNEB kit uses glass beads. The sample processing was conducted according to the manufacturers’ instructions with minor modifications (see “Materials and methods” for details). The obtained DNA samples were analyzed for various DNA characteristics and subjected to 16S rRNA sequencing (**Figure 1**). In addition, negative control samples without input materials were processed to estimate the “kitome” or the contamination that could be introduced by the DNA purification procedure itself. For each characteristic, the kits were ranked to allow a cross-comparison, so the lowest and the highest ranks correspond to the best and the worst performance, respectively.

#### DNA yield and quality

**Figure 3:**
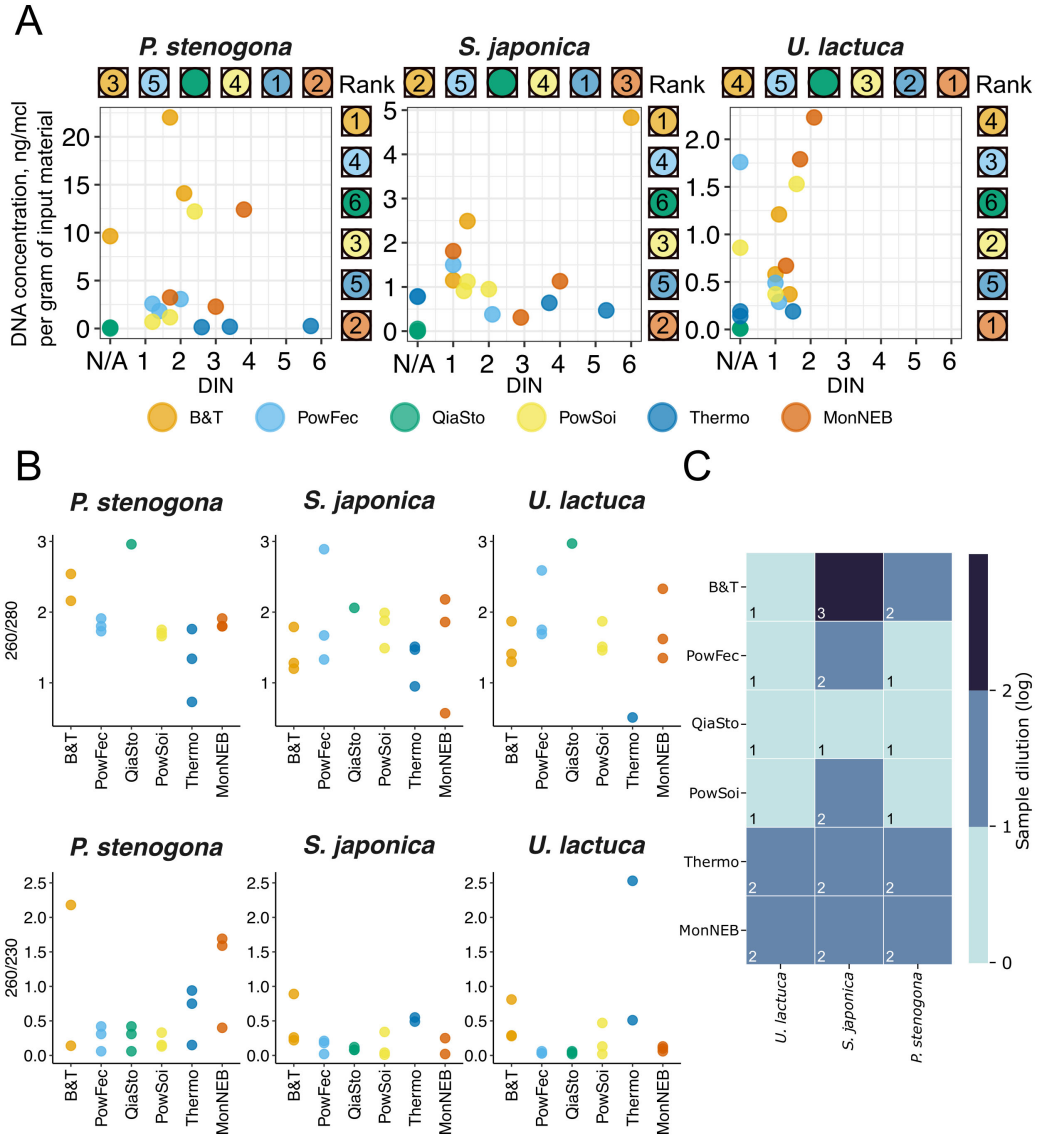
Comparison of DNA yield and purity across six commercial DNA extraction kits. **(A)** DNA concentrations and DNA integrity number (DIN) values. Data for three technical replicates are shown. N/A on the x-axis indicates samples where DIN could not be measured on TapeStation due to low DNA quality. Kit ranks are shown for DNA concentration (right) and DIN (top) of each subplot; lower ranks indicate higher values. QiaSto samples lack DIN ranks due to the DIN measurement failure for all samples. **(B)** DNA purity assessed by 260/280 (top) and 260/230 (bottom) absorption ratios. Data for three technical replicates are shown. **(C)** Presence of PCR inhibitors, estimated as a dilution factor required to achieve visible PCR amplification of the 16S rRNA gene region. The average of three technical replicates is shown. Kit ranks are shown in the lower right corner of heatmap cells; lower rank indicates lower sample dilution.

Similar to previous results (**Figure 2A**), *P. stenogona* samples produced the highest yields of DNA, while *S. japonica* and *U. lactuca* yields were rather low (*p* < 0.05, ANOVA) (**Figure 3A**, **Supplementary Table S2**). B&T, MonNEB, and PowSoi kits produced better results across three species, while QiaSto DNA yield was close to zero, limiting the application of this kit for macroalgae samples.

To estimate the fragmentation of DNA, the DNA integrity number (DIN) of the obtained samples was measured. It is widely considered that DIN of 6 and higher is acceptable for long-read sequencing, such as ONT or PacBio (Demkina et al., 2023). Correct estimation of DIN value is challenging for the samples with DNA concentration below 5 ng/μL, and thus the DIN values for *S. japonica* and *U. lactuca* could be compromised despite the efforts to concentrate these samples (**Figure 3A**). However, a visual inspection of capillary electropherograms revealed that while all samples of *S. japonica* (except those extracted with QiaSto) contained a high-molecular-weight (HMW) fraction, DNA extracted from *U. lactuca* was significantly degraded (**Figure 3A**, **Supplementary Figure S5**). The only samples of *U. lactuca* that contained visible HMW fraction were extracted with the MonNEB kit (**Supplementary Figure S5**). As for the *P. stenogona* samples, the DIN values were also low, and the HMW fraction was evident only for the Thermo and MonNEB treatments (**Figure 3A**, **Supplementary Figure S5**). It could be concluded that low yield and high DNA fragmentation are significant problems that could limit the long-read sequencing of macroalgae-associated biofilms and could be associated with host-derived nucleases. While not completely overcome by tested methodologies, the best result was achieved with the MonNEB kit.

#### DNA purity and presence of PCR inhibitors

Next, the contamination of recovered DNA samples with protein (by 260/280 nm ratio) and organic impurities (by 260/230 nm ratio) was measured. While 260/280 ratio was largely acceptable for most treatment types, all samples showed a significant contamination with organic impurities (low 260/230 ratio), suggesting that additional purification might be needed to obtain a high-quality DNA (**Figure 3B**).

The samples were tested for the presence of PCR inhibitors using undiluted and 10×- or 100×-diluted samples (**Figure 3C**, **Supplementary Figure S6**). In contrast to the previous results with the PowSoi kit, many treatments resulted in the inability to amplify the 16S rRNA region in undiluted samples. In particular, MonNEB samples required a 10× dilution to amplify DNA from all macroalgae species, while B&T samples, which had high DNA concentration, required a 10× dilution for *P. stenogona* and a 100× dilution for *S. japonica*. Among others, PowSoi and PowFec kits produced DNA that was suitable for direct amplification for two out of three macroalgae species, while all three QiaSto samples did not require dilution (**Figure 3C**, **Supplementary Figure S6**). The observed difference in amplification efficiencies between distinct macroalgae species could be explained by the different content of polysaccharides as the main inhibitors of PCR in macroalgae extracts. A cell wall of *S. japonica* (brown macroalgae) contains up to 40% of alginates of their dry weight (Lozada et al., 2022), which could inhibit downstream enzymatic reactions.

### Contamination levels and determination of “kitomes”

Sequencing of non-sample-specific contaminants, introduced during DNA purification from a kit’s reagents (also known as “kitome”), is a well-known problem in environmental metagenomics, especially for low-concentration DNA samples (Demkina et al., 2023; Olomu et al., 2020). To evaluate the “kitome” of different tested DNA extraction kits, DNA from no-input negative controls was isolated in parallel with macroalgae samples, after which the composition of “kitomes” and macroalgae biofilm samples from all three species purified with each kit was analyzed using 16S rRNA gene metagenomics.

Most samples isolated from *P. stenogona* and *U. lactuca* were sequenced to saturation, while *S. japonica* samples typically acquired fewer 16S reads (*p* < 0.01, ANOVA) (**Supplementary Figure S7**). Such differences may be attributed to the inhibition of PCR or other enzymatic reactions involved in the sequencing library preparation process in *S. japonica* samples. Interestingly, *S. japonica* samples obtained with the QiaSto kit acquired a normal amount of 16S reads, which correlates with the PCR inhibition assay results (**Figure 3C**) and indicates that this kit is an efficient solution for the removal of PCR inhibitors. Poor sequencing results obtained with the majority of DNA extraction kits for *S. japonica* highlight the need for additional DNA purification steps before sequencing of metagenomic DNA obtained from this species.

The number of reads obtained for “kitomes” varied significantly between DNA extraction kits. B&T, PowFec, and Thermo “kitomes” had particularly high read yields comparable to macroalgae samples, which could possibly cause contamination issues (**Supplementary Figure S8D**, **Supplementary Table S4**). The ratio between the yields of macroalgae reads and “kitome” reads (M/C ratio) was above 1 in the case of QiaSto, PowSoi, and MonNEB kits (**Supplementary Table S4**). The QiaSto kit demonstrated the highest ratio, a consistently high yield of macroalgae reads, and a low yield of “kitome” reads (**Supplementary Figure S8D**, **Supplementary Table S4**).

**Figure 4:**
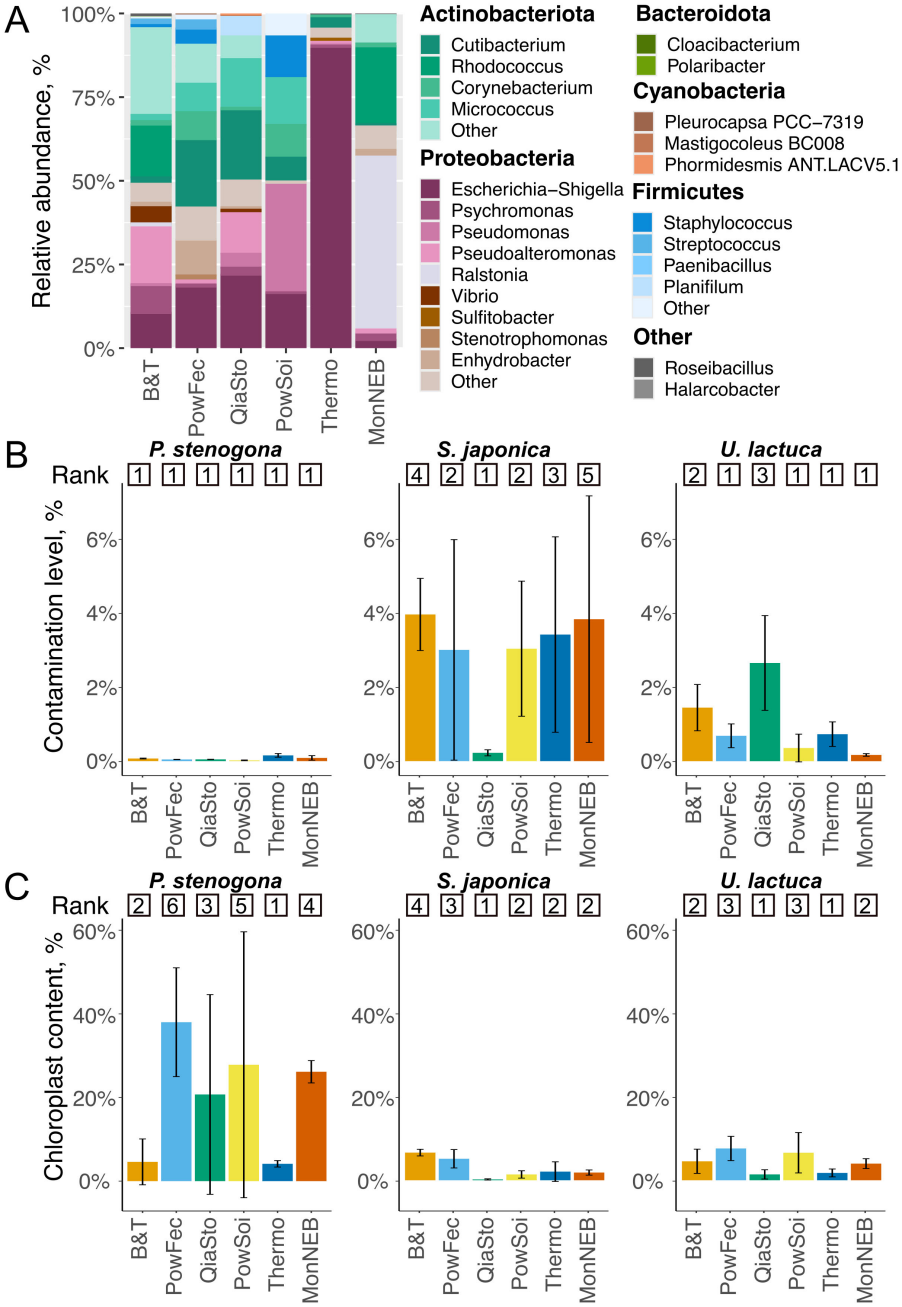
Analysis of “kitomes” and contamination levels of samples extracted with different kits. **(A)** Relative abundance of microbial orders in “kitomes” (laboratory control groups). Bacterial genera with relative abundances >1% are shown. **(B)** Relative levels of contamination for different groups of samples. Bars and error bars represent mean relative contamination and SD for three biological replicates. Numbers above represent kit ranking. Lower rank indicates lower sample contamination. **(C)** Relative levels of chloroplast content. Bars and error bars represent mean relative contamination and SD for three biological replicates. Numbers above represent kit ranking. Lower rank indicates lower sample contamination.

The “kitome” samples were dominated by bacterial genera known as human-associated and/or highlighted as potential kit contaminants previously—*Staphylococcus* and *Streptococcus* (Firmicutes/Bacillota), *Cutibacterium*, *Corynebacterium*, and *Micrococcus* (all from Actinobacteriota), *Escherichia*, *Pseudomonas*, and *Ralstonia* (all from Gammaproteobacteria) (Salter et al., 2014). “Kitomes” of different kits had relatively similar compositions, except that *Escherichia* was enriched in Thermo and *Ralstonia* was enriched in MonNEB “kitomes” (**Figure 4A**). The total abundance of contaminant OTUs in macroalgae samples did not exceed 2%–4% for *S. japonica* and *U. lactuca* samples and was significantly lower for *P. stenogona* samples (*p* < 0.05, ANOVA), indicating that a lower concentration of sample DNA could result in an increased proportion of contaminant OTUs (**Figure 4B**, **Supplementary Figure S8A**). These values are comparable to the contamination levels observed previously for freshwater and marine sediments (Demkina et al., 2023) and are common for samples rich in microbial biomass.

It was noticed that a considerable fraction of 16S reads originated from chloroplasts (**Figure 4C**, **Supplementary Figure S8B**) and universal bacterial 16S primers can amplify conserved 16S genes from plastid genomes (Artimová et al., 2022). This allowed us to estimate the contamination of samples with eukaryotic DNA using the abundance of chloroplast OTUs as a proxy. *S. japonica* and *U. lactuca* datasets typically contained 5%–10% of chloroplast OTUs, while *P. stenogona* contained up to 40%, indicating a species-specific pattern of host DNA contamination. Samples obtained using Thermo and QiaSto kits tend to be less contaminated with chloroplast reads and acquire the best ranks. In contrast, the usage of PowFec and PowSoi kits was associated with increased levels of chloroplast reads, and these kits were ranked the worst (**Figure 4C**, **Supplementary Figure S8B**). Considering contamination with “kitome” and chloroplast sequences, any kit could be consistently top-ranked (**Supplementary Figure S8C**). The performance of the kits was rather species-specific.

### Effects of DNA extraction kits on the composition of bacterial communities

**Figure 5:**
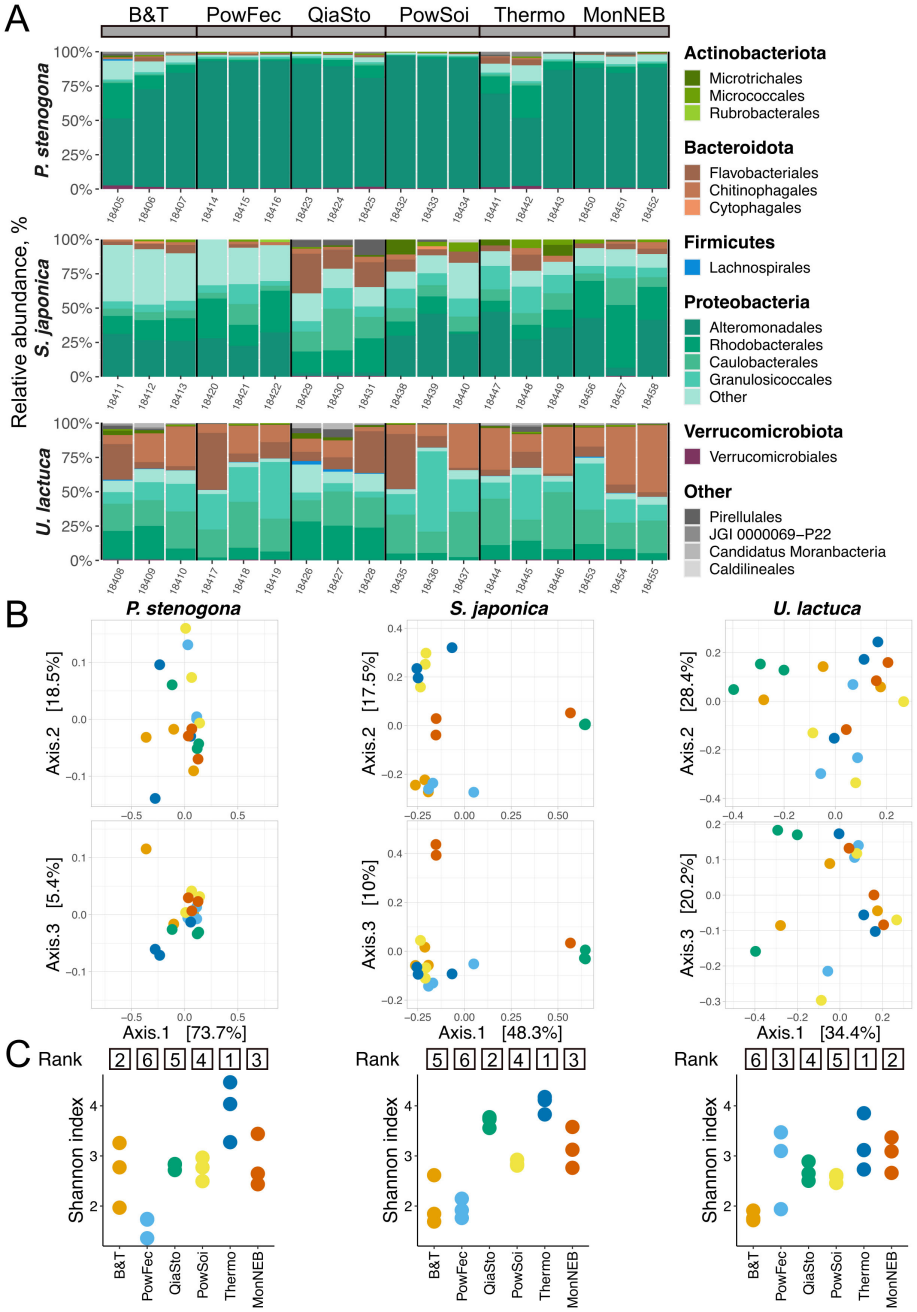
Effect of DNA extraction kit on microbiome composition and diversity. **(A)** Relative abundance of bacterial orders after contamination removal with Decontam. Bacterial orders with relative abundances >1% are shown. **(B)** PCoA of Bray–Curtis dissimilarities for different macroalgae species and projections of principal components (PC) 1 and 2 (top) and PC1 vs PC3 (bottom). **(C)** Shannon index values of macroalgae samples for *P. stenogona* (left), *S. japonica* (middle), and *U. lactuca* (right). Numbers above indicate kit ranking by alpha-diversity; lower rank indicates a higher Shannon index. Data on all panels are shown for three technical replicates.

After the removal of contaminating “kitome” and chloroplast OTUs, kit-specific biases in the composition of bacterial communities associated with macroalgae were evaluated (**Supplementary Figure S9**). *P. stenogona* samples had a consistent composition across methods, with a slight deviation observed for B&T and Thermo kits (**Figures 5A, B**). The latter kits were associated with elevated abundance of Flavobacteriales (Bacteroidota) and Rhodobacterales (Proteobacteria). Notably, these kits had the lowest mean read counts, which may impact the microbial composition (**Supplementary Table S4**). For *S. japonica*, samples were clustered by the amount of acquired reads, with samples processed using the QiaSto kit and a single replicate obtained with the MonNEB kit separated from other samples. These samples had a decreased abundance of Altermononadales and an increased abundance of Caulobacterales (both Proteobacteria). *U. lactuca* samples had relatively low compositional differences among the kits. Only QiaSto samples and two out of three samples processed with the B&T kit slightly deviated from other samples and had an increased level of Rhodobacterales (Proteobacteria). Using the PERMANOVA method, kit selection was found to be a factor significantly affecting the composition of microbial communities for *S. japonica* and *U. lactuca* (*p*-values < 0.05, PERMANOVA).

Though the ground-truth composition of microbiomes is not known, it can be considered that increased alpha-diversity may reflect better sampling. Following this logic, Thermo and MonNEB kits were top-ranked by the Shannon alpha-diversity index for all tested macroalgae species (*p* < 0.05, Kruskal–Wallis test) (**Figure 5C**), suggesting better composition capturing. In contrast, samples obtained using B&T, PowFec, and PowSoi kits typically had decreased alpha-diversity (*p* < 0.05, Kruskal–Wallis test).

### Technical reproducibility of DNA extraction kits

Technical reproducibility of DNA extraction is an important characteristic that may critically affect the revealed composition of microbial communities and subsequent conclusions. To assess the reproducibility of tested kits, an approach previously implemented for comparing widely used DNA extraction kits was used, in which the reproducibility rate was calculated as a fraction of OTUs shared between all technical replicates (Demkina et al., 2023).

**Figure 6:**
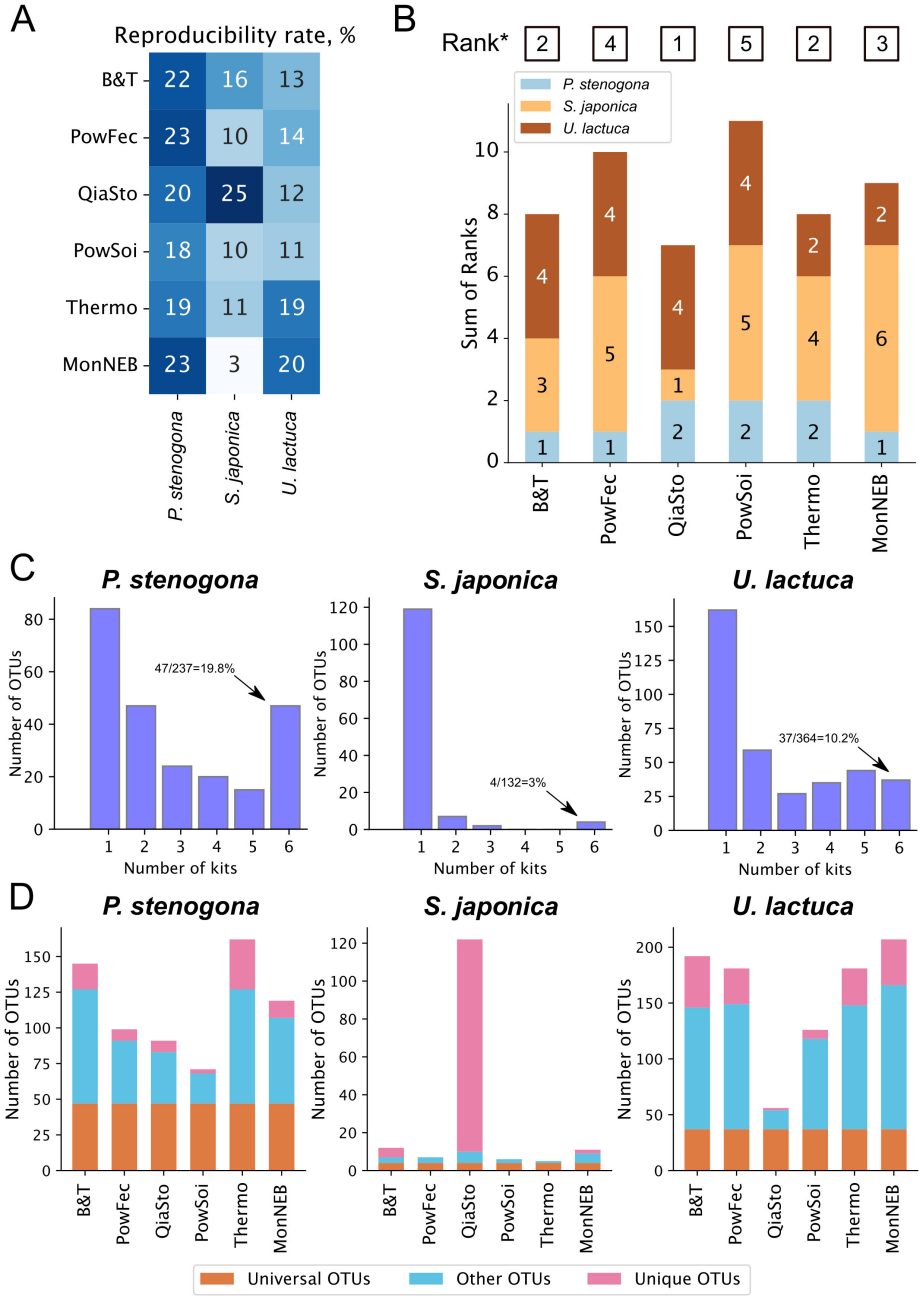
Reproducibility of microbiome composition across DNA extraction kits. **(A)** Reproducibility percentages for each kit across macroalgae species. **(B)** Ranking of DNA extraction kits by their reproducibility percentage on **(A)**. On each bar, kit rank is shown, with different colors specifying different algal species. Lower rank indicates a higher reproducibility percentage. (*)—kit ranking is based on the sum of ranks for all algal species. **(C)** Numbers of OTUs shared between different numbers of DNA extraction kits tested; from 1 (OTU was found in a single sample processed with a single kit) to 6 (OTU was found in all samples processed with any of the tested kits). **(D)** Fractions of OTUs: universal (found in all kits, orange), unique (found in a single kit, pink) OTUs found by just one kit (unique OTUs, pink), or shared by more than one but not all kits (other OTUs, light blue) for each algal species.

It was found that reproducibility rates were species-specific, with higher rates observed for *P. stenogona* and lower rates associated with *S. japonica* samples (**Figure 6A**). *P. stenogona* samples were consistently processed by all kits, with slightly higher reproducibility rates observed for MonNEB, PowFec, and B&T kits. Almost 20% of OTUs (47 OTUs) identified for this type of sample were captured by all DNA extraction kits (**Figure 6C**), which is a typical rate observed previously for samples rich in microbial load (Demkina et al., 2023). The reduced reproducibility detected in *S. japonica* samples might be attributed to lower DNA yields or inhibitors of the PCR reaction. Consistently, only four OTUs (3% of all detected bacterial OTUs for this macroalgae species) were shared among all DNA isolation conditions (**Figure 6C**). In line with this hypothesis, a high (actually, the highest observed across all kits and all species) reproducibility rate in *S. japonica* samples was observed for the QiaSto kit, which was shown to efficiently remove PCR inhibitors (**Figure 3C**). Using the QiaSto kit, a considerably more diverse community was detected, with the majority of OTUs present only in these samples (**Figure 6D**, **Supplementary Figure S10**). It can be speculated that a community composition revealed with the QiaSto kit might be the most accurate representation of the actual *S. japonica*-associated microbiome across the kits tested. *U. lactuca* samples had intermediate reproducibility rates between the other two macroalgae species. Interestingly, Thermo and MonNEB kits showed higher reproducibility rates with *U. lactuca* samples than other kits. Overall, QiaSto, B&T, and Thermo kits had better reproducibility across all samples and were top-ranked by this parameter (**Figure 6B**). In contrast, PowFec and PowSoi underperformed and acquired the worst total ranks.

## Discussion

Marine macroalgae are a vital component of the ocean ecosystem (Lee et al., 2013). They have numerous applications, including medicine (Cornish and Garbary, 2010; Kasanah et al., 2015; Kim et al., 2019; Lins et al., 2009; Sørensen et al., 2019), food (Mouritsen et al., 2013), biotechnology and cosmetics (Cian et al., 2018; Corey et al., 2012; Kazir et al., 2019), renewable energy (Demirbas, 2010), and biofuel (Kumar et al., 2013). Microbial communities associated with macroalgae play an important role in their health and development (Gachon et al., 2010). Extraction of metagenomic DNA from macroalgae samples remains a challenging task. Macroalgae biofilms contain high levels of phenolic compounds and polysaccharides such as laminaran, alginate, and fucoidan, which can inhibit enzymatic reactions upon DNA extraction (Pearman et al., 2024; Skriptsova et al., 2012). The microbiome of macroalgae can also be affected by environmental factors such as season, temperature, salinity, nutrient availability, depth, and location on a macroalgae thallus (Krieg, 1996).

The results of any metagenomic analysis are dependent on the quality of the obtained DNA. Each step of the DNA extraction process can introduce its own biases, which can influence the accuracy of the final data analysis. Although numerous studies describe the selection of methods for sample preparation and DNA extraction from the human microbiome (Panek et al., 2018; Wagner Mackenzie et al., 2015; Yuan et al., 2012) or specific microbial communities (Corcoll et al., 2017; Ketchum et al., 2018), benchmarking of DNA extraction methods for marine samples, particularly macroalgae thalli, is scarce.

To address this challenge, five methods for bacterial cell recovery from the surface of macroalgae thalli and six commercially available DNA extraction kits were compared using samples from macroalgae species representing three major groups: *Saccharina japonica* (brown algae/Phaeophyceae), *Ulva lactuca* (green algae/Chlorophyta), and *Palmaria stenogona* (red algae/Rhodophyta) (**Figure 1**). The first phase of the study involved testing several bacterial cell recovery methods with one kit (PowSoi). Incubation of macroalgae thalli with a buffer containing a detergent and enzyme mixture was shown to increase the recovery of cells from biofilms while avoiding problems of whole-sample homogenization that result in high eukaryotic DNA loads. In the second phase of this study, different kits were tested with samples obtained with a single cell recovery method (incubation with a mix of PBS with 1% BFR). Various DNA characteristics were evaluated, including DNA quantity (concentration), purity (260/280 and 260/230 absorption ratios), fragmentation (DNA integrity, DIN), presence of PCR inhibitors, as well as characteristics of a microbial community composition, such as alpha- and beta-diversity, contamination level, and reproducibility of metagenomic sequencing results. Based on these parameters, DNA purification kits were ranked according to their efficiency.

Among the other factors that should be considered when selecting a DNA isolation method are simplicity, accessibility, and protocol duration. An advantage of microbial cells’ recovery via incubation is the reduced amount of contaminant eukaryotic DNA, while overall DNA yields are decreased compared with mechanical cell recovery methods such as swabbing or homogenization. On the other hand, homogenization is significantly faster compared to other methods. The main disadvantage of swabbing is its labor intensity, while a significant admixture of eukaryotic DNA and contaminants was also observed in this study. Given these factors, it is not possible to identify the most convenient cell recovery method (**Table 2**). The selection of a method should be guided instead by specific goals of the study and the type of macroalgae.

**Table 2:** Comparison of bacterial cell recovery methods by three major characteristics: disruption of eukaryotic cells/contamination with eukaryotic DNA, relative simplicity (where 1 is for the simplest method, and 5 is for the most complex and labor-intensive method), and duration of a protocol.

Bacterial cells recovery method	Evident host cells disruption	Simplicity (1–5)	Time
PBS	No	1	2–2.5 h
PBS + 0.1% BFR	No	1	2–2.5 h
PBS + 1% BFR	No	1	2–2.5 h
Swabbing	Yes	5	15–30 min
Homogenization	Yes	3	5–10 min

Based on DNA yields, homogenization was the most efficient method for recovery of bacterial cells from macroalgae thalli. However, this may be associated with the disruption of macroalgae cells and contamination with the host DNA. Homogenization promotes the release of organic impurities (such as polysaccharides) that can inhibit downstream enzymatic reactions or adsorb DNA during purification (Schrader et al., 2012; Skriptsova et al., 2012). It was noticed that bacterial cell removal with swabbing also resulted in damaging the host cells. Bacterial cell recovery via incubation with a buffer, while less efficient, could be improved by the addition of detergents and enzymes acting on the biofilm matrix, resulting in decreased contamination with the host material and PCR inhibitors. It should also be noted that DNA yield varies depending on the macroalgae species. The poorest results were obtained for *S. japonica*, and this may be explained by the chemical composition of *Saccharina*’s specific extracellular matrix (Mizuta and Yasui, 2012).

**Figure 7:**
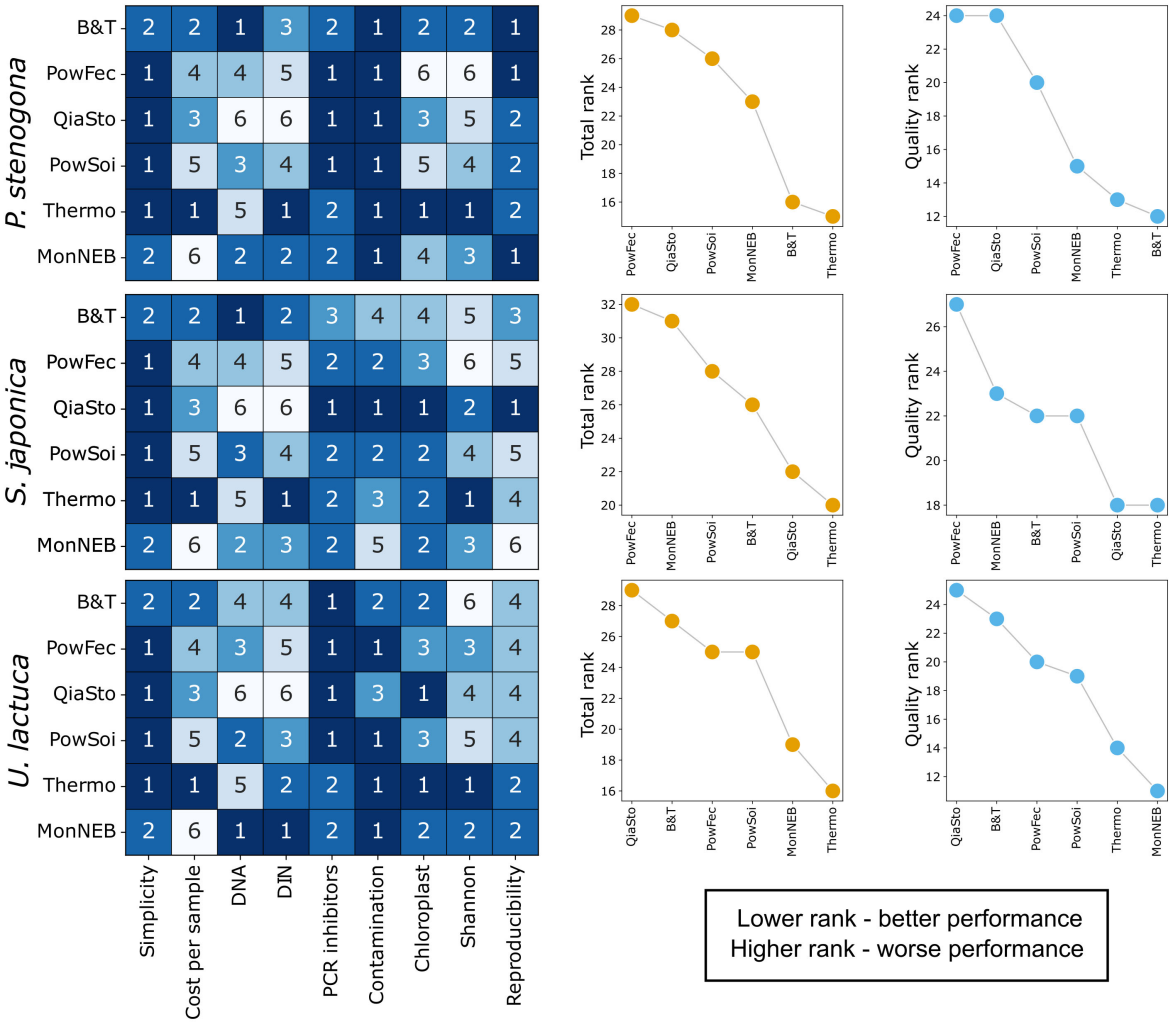
Resulting ranking of the DNA extraction kits tested. Each row represents a macroalgae species with kits ranked exclusively for that species. Each row consists of a heatmap representing kit ranks obtained for different comparison parameters (DNA—DNA concentration obtained from the sample, DIN—DNA integrity number) on the left; summed and sorted kit ranks for different macroalgae species (Total rank) in the middle; summed and sorted kit ranks excluding the simplicity and cost-per-sample ranks (Quality rank) on the right. Lower rank indicates better performance.

Following the benchmarking scheme, six DNA extraction kits were evaluated based on their simplicity (the ease of use) as well as price per sample (see **Table 3**). All kits presented in this study are relatively convenient for DNA extraction. For B&T, a specific selection of a lysis buffer was required, while the glass beads implemented in the MonNEB kit require more careful handling compared to the spin columns used in other kits. In addition, MonNEB was the most expensive across all tested kits. The Thermo kit was the least expensive option evaluated. When cost and protocol simplicity were included in the ranking metrics, this kit showed the best overall performance across all algal species (**Figure 7**), suggesting that it may serve as a practical universal choice under resource-limited conditions. However, several limitations should be noted: DNA yields obtained with this kit were rather low for all species, and it exhibited moderate contamination levels and low reproducibility for *S. japonica*. When cost and simplicity were excluded from the ranking analysis, the Thermo kit still remained among the top performers for all species (**Figure 7**). Under these conditions, the B&T kit ranked highest for *P. stenogona*, QiaSto and Thermo shared the top position for *S. japonica*, and the MonNEB kit performed best for *U. lactuca*.

**Table 3:** Comparison of DNA extraction kits by five major characteristics: method for bacterial cells lysis (“lysis method”), method of DNA extraction and purification (“DNA extraction”), relative simplicity (where 1 is for the most simple kit, and 5 is for the most complex and labor-intensive kit), cost per one package ($1 = €0.92), and cost per one sample.

Kit	Lysis method	DNA extraction method	Simplicity (1–5)	Cost per sample
PowSoi	Mechanical, chemical	Spin column	1	$9.8
PowFec	Mechanical, chemical	Spin column	1	$9.66
QiaSto	Heat, chemical	Spin column	1	$7.68
B&T	Heat, chemical + enzymatic	Spin column	2	$4.48
Thermo	Heat, chemical + enzymatic	Spin column	1	$3.9
MonNEB	Heat, mechanical + enzymatic	Glass beads	2	$10.78

This study did not identify any particular kit capable of producing high-quality DNA immediately suitable for long-read sequencing, i.e., the DNA with high concentration, low fragmentation, minimal PCR inhibitors content, and low contamination with host DNA. It was rather found that kits generating higher yields of DNA tend to be less efficient at removing PCR inhibitors—for example, the B&T kit, which was top-ranked by DNA yield, recovered DNA of an average quality that was not directly suitable for 16S rRNA PCR amplification. Similarly, the MonNEB kit, also ranked among the top-performing kits by DNA yield, integrity, and purity, was particularly prone to carryover of PCR inhibitors. Thus, additional downstream purification steps could be recommended while working with these kits. A similar trade-off between DNA yield and purity can be illustrated by QiaSto kit performance. The QiaSto kit produced lower DNA yields; however, it was among the top performers in terms of DNA purity and effectively removed PCR inhibitors. Consequently, the purity of QiaSto samples translated to an increased reproducibility rate for this kit as was assessed by 16S metagenomics. This kit could be recommended for macroalgae samples with an increased level of enzymatic inhibitors.

PowSoi and PowFec kits are frequently considered as “gold standards” for a diverse set of samples (Child et al., 2024; Demkina et al., 2023; Shaffer et al., 2022). In the current benchmark, they demonstrated medium performance in DNA yield and integrity and provided a higher-than-average level of purity but showed an increased contamination with chloroplast OTUs and reduced alpha-diversity and reproducibility of microbiome composition. Overall, these kits underperformed (PowFec) or were ranked in the middle (PowSoi) in aggregated ranks for all tested characteristics, including or excluding the cost and simplicity (**Figure 7**). Such a result indicates that other commercial DNA extraction kits can be considered as a good alternative for the “gold standard” kits, supporting recent observations made for different types of environmental samples (Demkina et al., 2023; Sobolev et al., 2025).

This study has several inherent limitations. The analysis was conducted on a limited number of algal species from a single geographic location and time point, which may not capture the full variability of biofilm communities influenced by seasonality, environmental conditions, or algal health status. Compared to previous studies (Demkina et al., 2023; Roager et al., 2023; Sobolev et al., 2025), the DNA quality and yield in this study generally were low, particularly for *S. japonica* and *U. lactuca*. This indicates that the extracted DNA may still be suboptimal for long-read sequencing technologies, which require high-molecular-weight DNA and could affect the conclusions about the abundance of various taxa, along with low read counts after 16S rRNA sequencing. The presence of chloroplast OTUs, despite reflecting eukaryotic DNA presence, can diminish the number of reads remaining after filtering chloroplasts from the data. A further significant constraint is the absence of a known “ground truth” microbial community composition; consequently, although the consistency and diversity revealed by each method were assessed during this study, it is not possible to definitively determine which protocol most accurately reflects the true *in situ* community—only which is most effective and reproducible within the constraints of the experimental setup. These limitations highlight the need for future studies that incorporate mock communities and broader sampling to validate and refine these methodological recommendations.

## Conclusion

This study systematically benchmarked methods for recovering and sequencing the microbial communities associated with marine macroalgae, revealing a fundamental trade-off between DNA yield and purity. While whole-sample homogenization maximized DNA recovery, it introduced a significant contamination from host chloroplast DNA. In contrast, incubating algal thalli in a PBS buffer supplemented with a detergent–enzyme mixture (BFR) minimized host contamination while maintaining sufficient DNA yield, establishing it as the preferred cell recovery method.

The subsequent evaluation of six DNA extraction kits demonstrated that their performance was highly dependent on the algal species, with no single kit excelling in all metrics. The GeneJET Genomic DNA Purification Kit (Thermo Scientific) emerged as the most versatile overall solution. However, species-specific recommendations were identified, namely: QIAamp Fast DNA Stool Mini Kit was most effective for the polysaccharide-rich brown alga *S. japonica*, Monarch HMW DNA Extraction Kit was superior for the green alga *U. lactuca*, and DNeasy Blood & Tissue Kit performed best for the red alga *P. stenogona*. This comparative benchmark provides a critical foundation for selecting optimized, species-appropriate protocols for metagenomic studies of algal microbiomes (**Figure 7**).

## Data Availability

The datasets presented in this study can be found in online repositories. The names of the repository/repositories and accession number(s) can be found below: https://www.ncbi.nlm.nih.gov/, PRJNA1271329.
