# Supplementary material for: Efficient recovery and DNA extraction for algae-associated microbial communities
Source: Front Plant Sci. 2026 Jan 5;16:1693747. doi: 10.3389/fpls.2025.1693747 (PMC12813104; doi:10.3389/fpls.2025.1693747)
Supplement: Supplementary file 1 [file DataSheet1.pdf]

Supplementary Figures

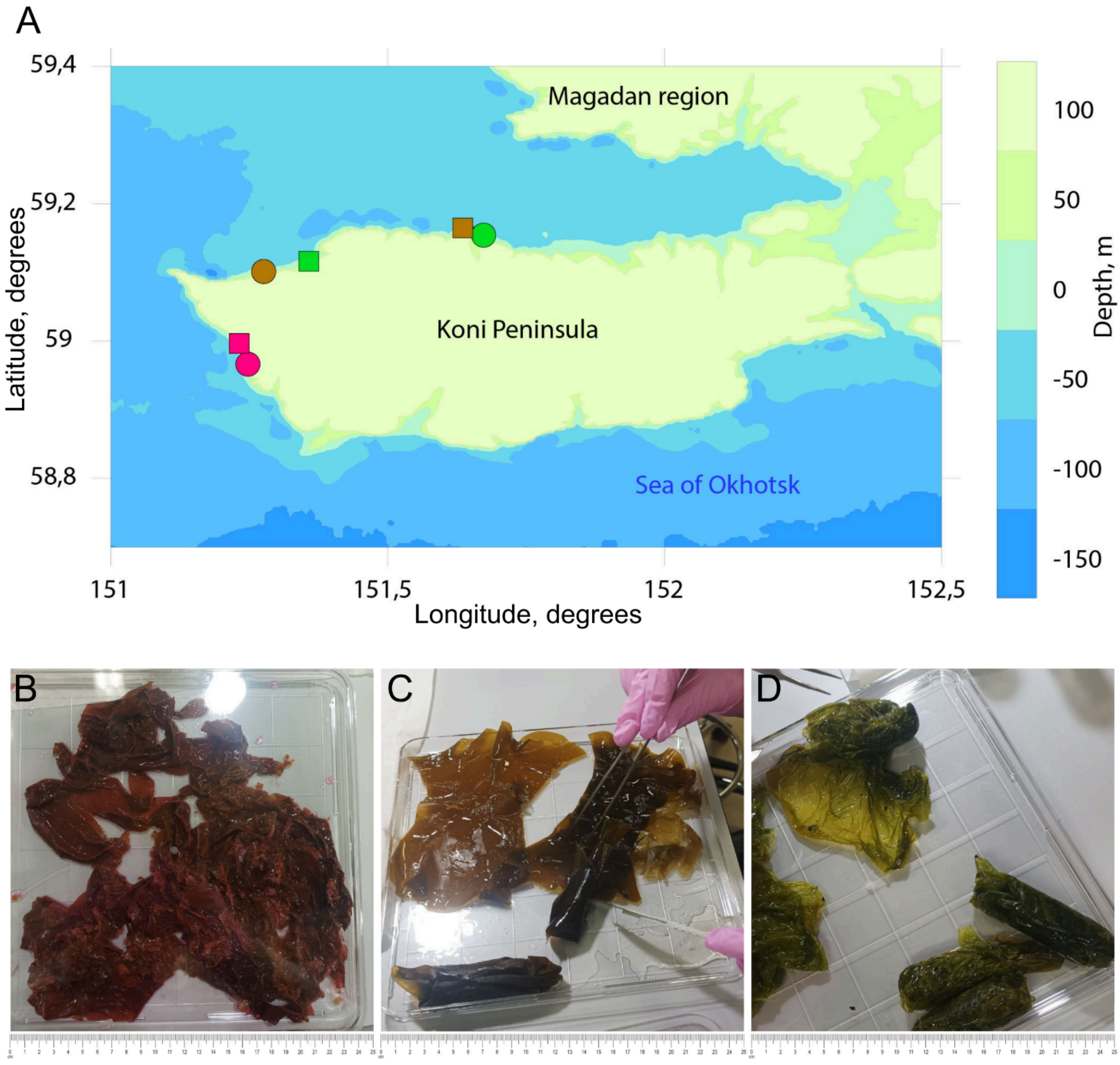

Supplementary Figure 1. Macroalgae samples analyzed in the study. (**A**) Geographical location of macroalgae sample collection sites. Squares - samples used for biofilm recovery comparison, circles - samples used for DNA purification kits comparison. Colors correspond to algae types: brown sites — *Saccharina japonica* (also known as brown algae or Phaeophycea), green sites — *Ulva lactuca* (or green algae, Chlorophycea), and red sites — *Palmaria stenogona* (or red algae, Rhodophycea). (**B-D**) Photos of the representative samples of macroalgae (**B** — *Palmaria stenogona*, **C** — *Ulva lactuca*, **D** — *Saccharina japonica*) after collection. A scale in cm is shown below the images.

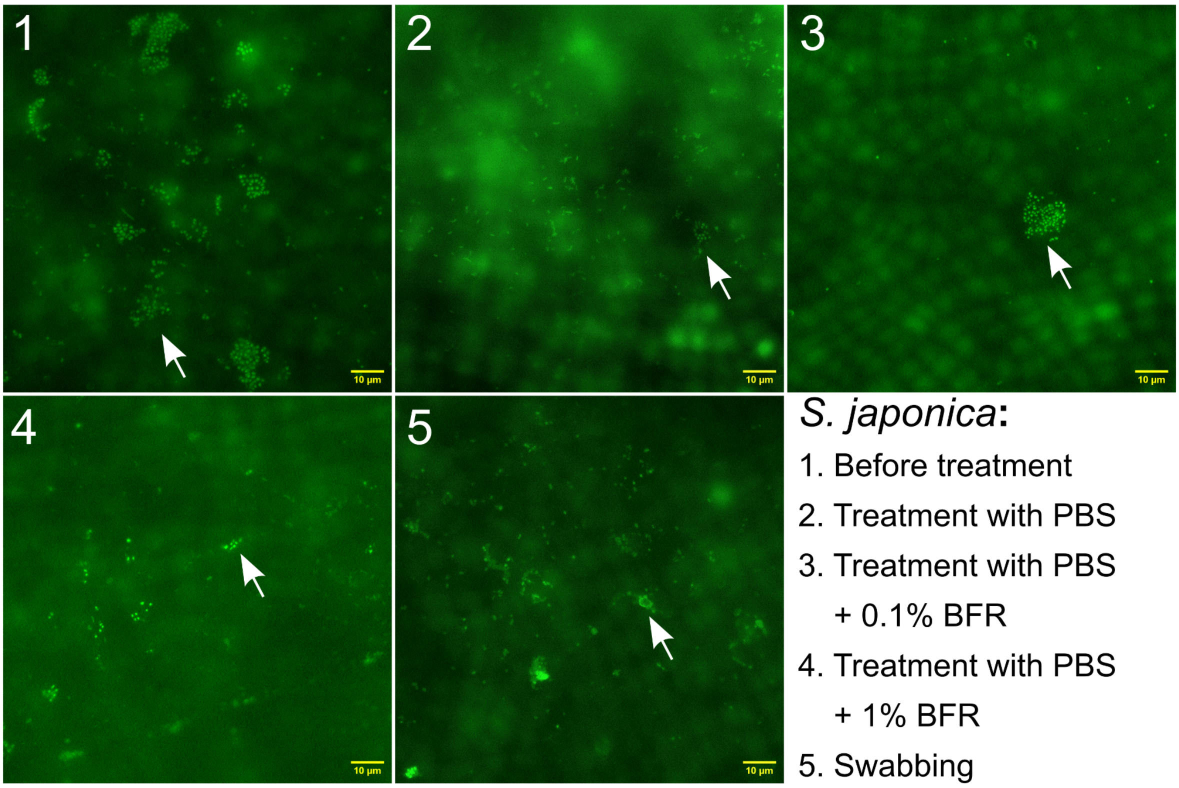

Supplementary Figure 2. Representative confocal microscopy images of *S. japonica* surface biofilm before (1) and after (2-5) exposure to various sample preparation methods. White arrows indicate examples of bacterial cells.

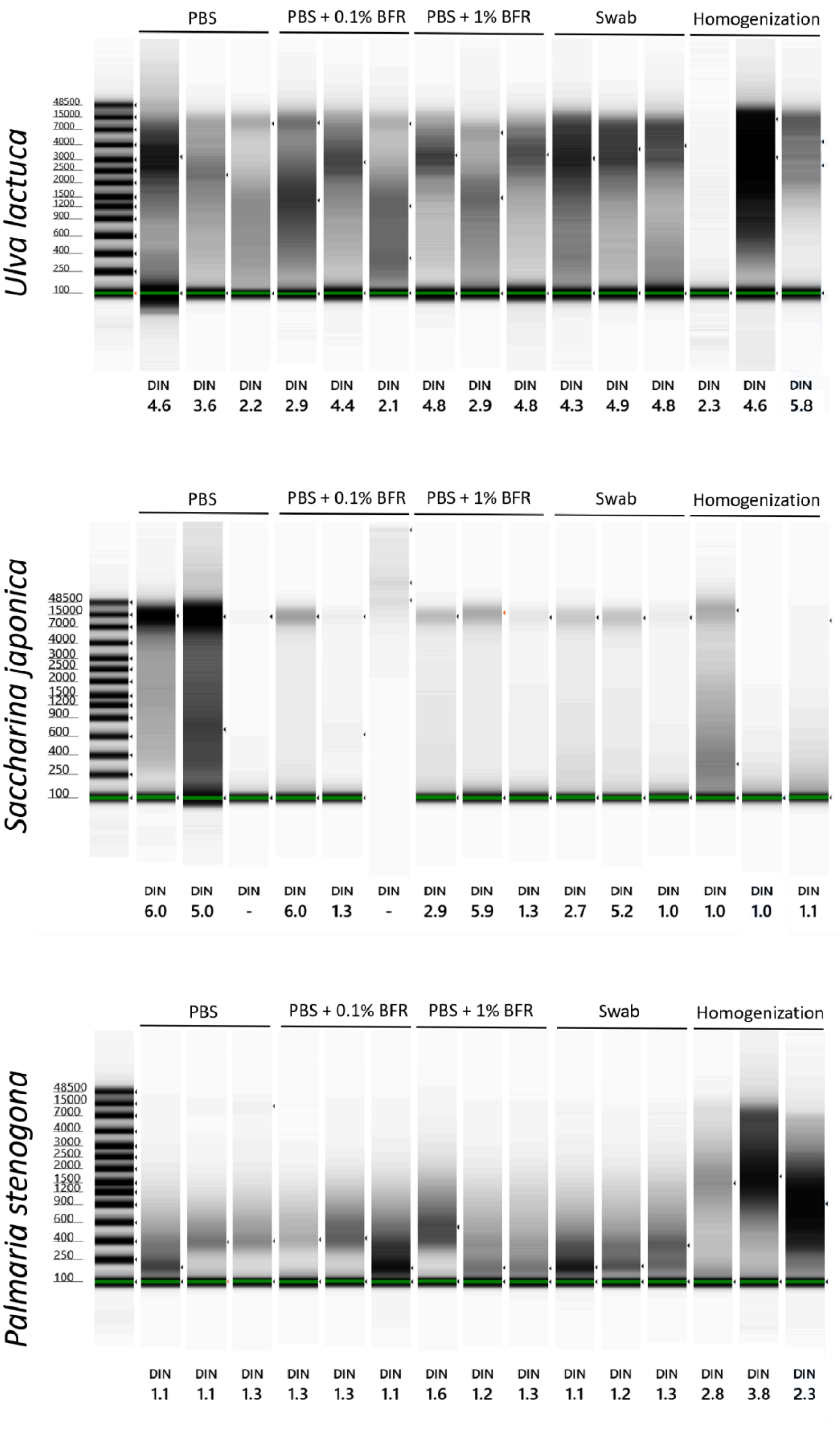

Supplementary Figure 3. DIN values for DNA samples obtained with different sample preparation methods for surface biofilms associated with three macroalgae species (**A** — *U. lactuca*; **B** — *S. japonica*; **C** — *P. stenogona*). DIN values were obtained by capillary electrophoresis performed on a TapeStation 4150 (Agilent) with Genomic DNA ScreenTape System.

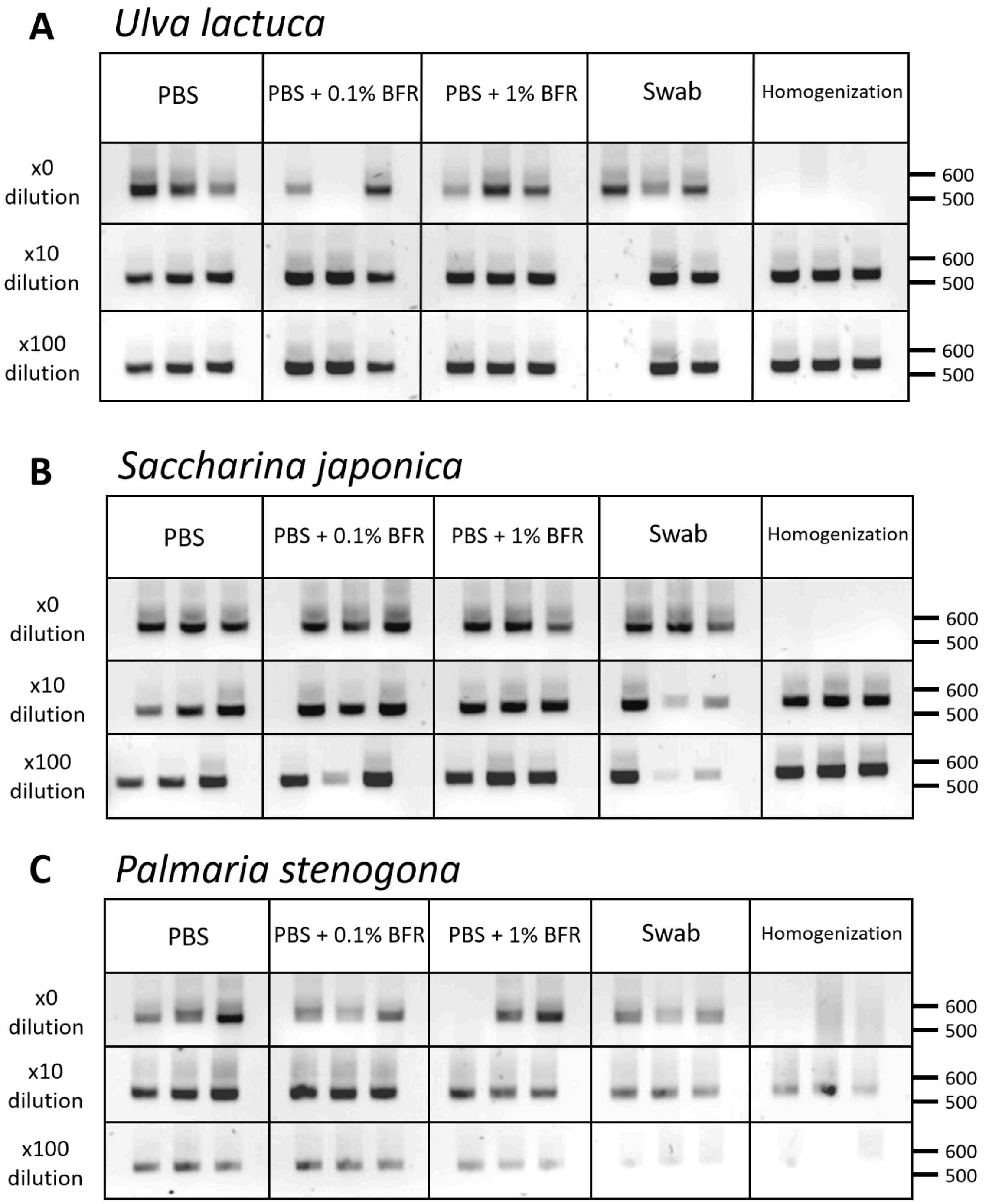

Supplementary Figure 4. Results of the 16S rRNA gene PCR for different types of sample preparation and macroalgae species: (**A**) — *U. lactuca*; (**B**) — *S. japonica*; (**C**) — *P. stenogona*). PCR was performed with non-diluted, 10- fold and 100-fold diluted input DNA.

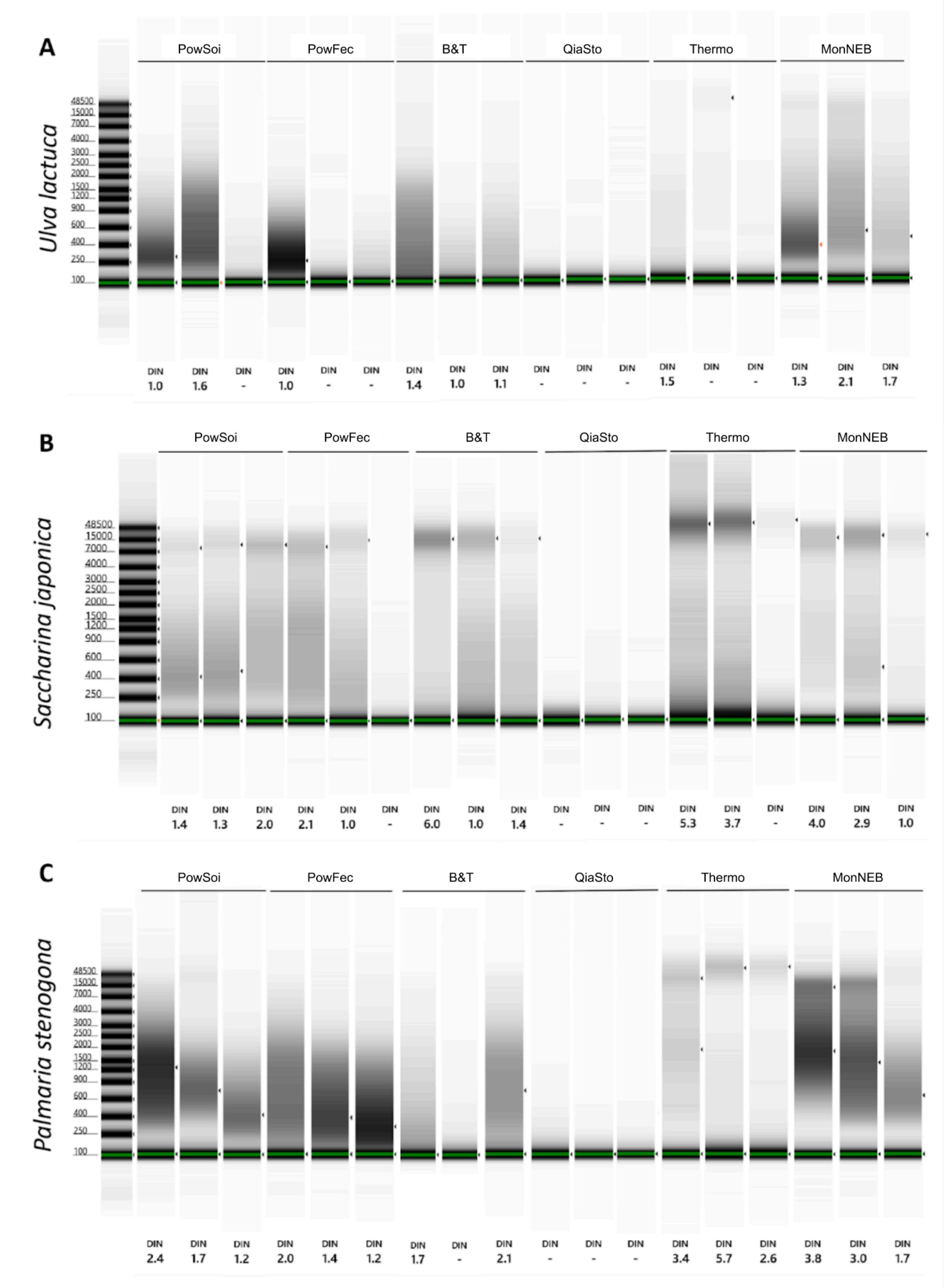

Supplementary Figure 5. DIN values for DNA samples obtained with different DNA extraction kits for surface biofilms associated with three macroalgae species: (**A)** — *U. lactuca*; (**B)** — *S. japonica*; (**C)** — *P. stenogona*). DIN values were obtained by capillary electrophoresis performed on a TapeStation 4150 (Agilent) with Genomic DNA ScreenTape System.

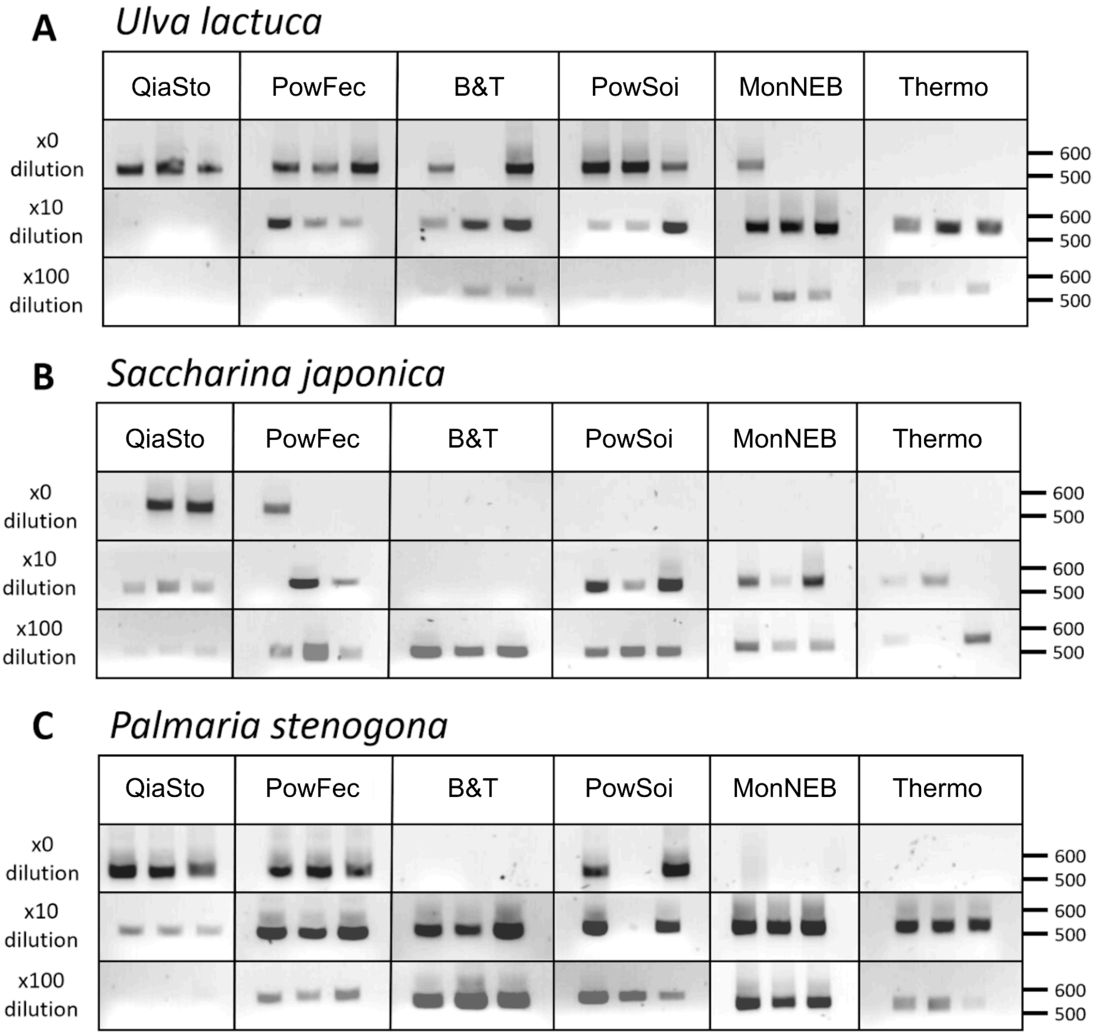

Supplementary Figure 6. Results of the 16S rRNA gene PCR for different kits and algae species: (**A**) — *U. lactuca*; (**B**) — *S. japonica*; (**C**) — *P. stenogona*). PCR was performed with non-diluted, 10- fold and 100-fold diluted input DNA.

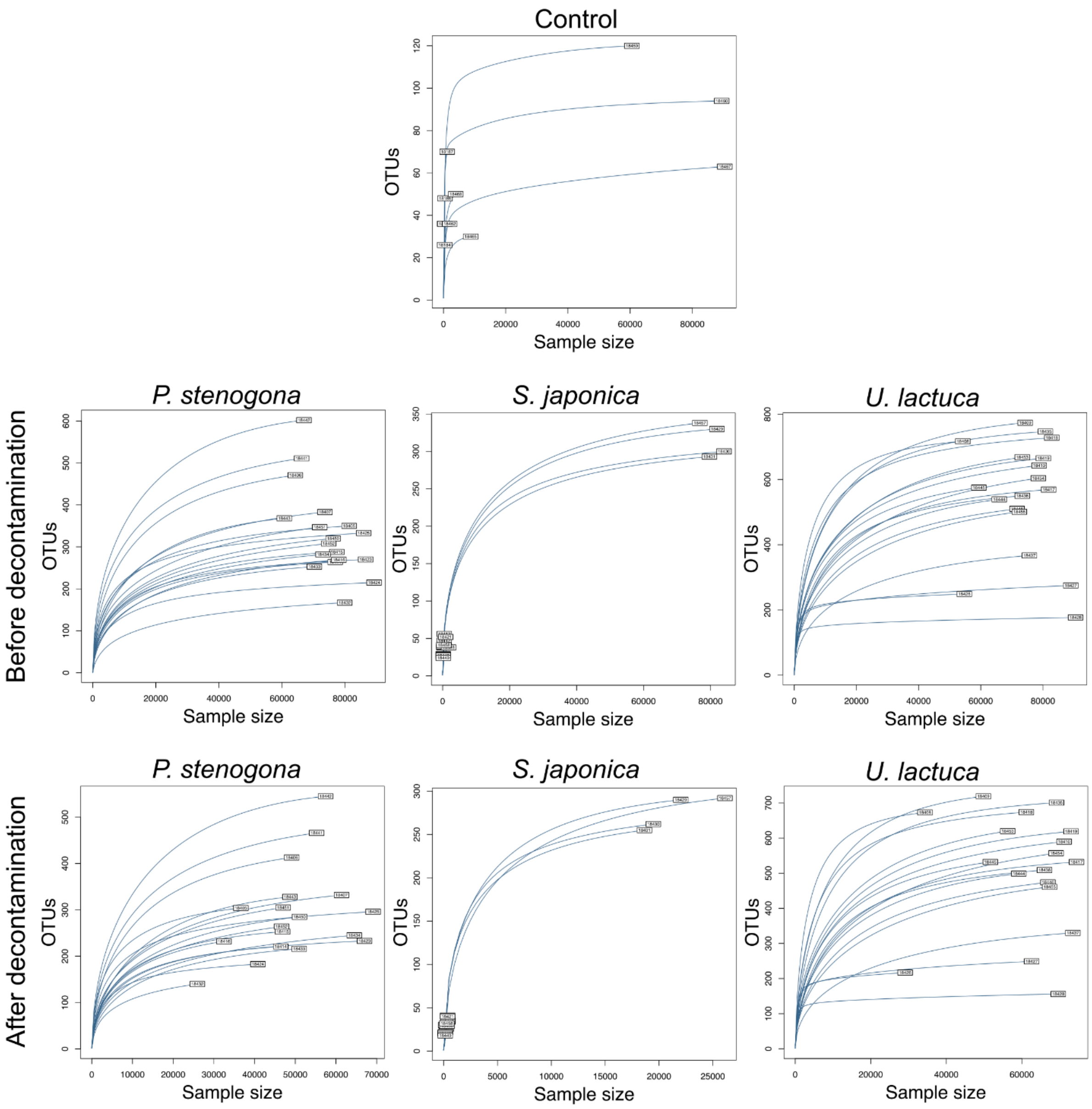

Supplementary Figure 7. Rarefaction curves of macroalgae samples. Top row: control samples. Middle row: macroalgae samples before decontamination. Bottom row: macroalgae samples after decontamination.

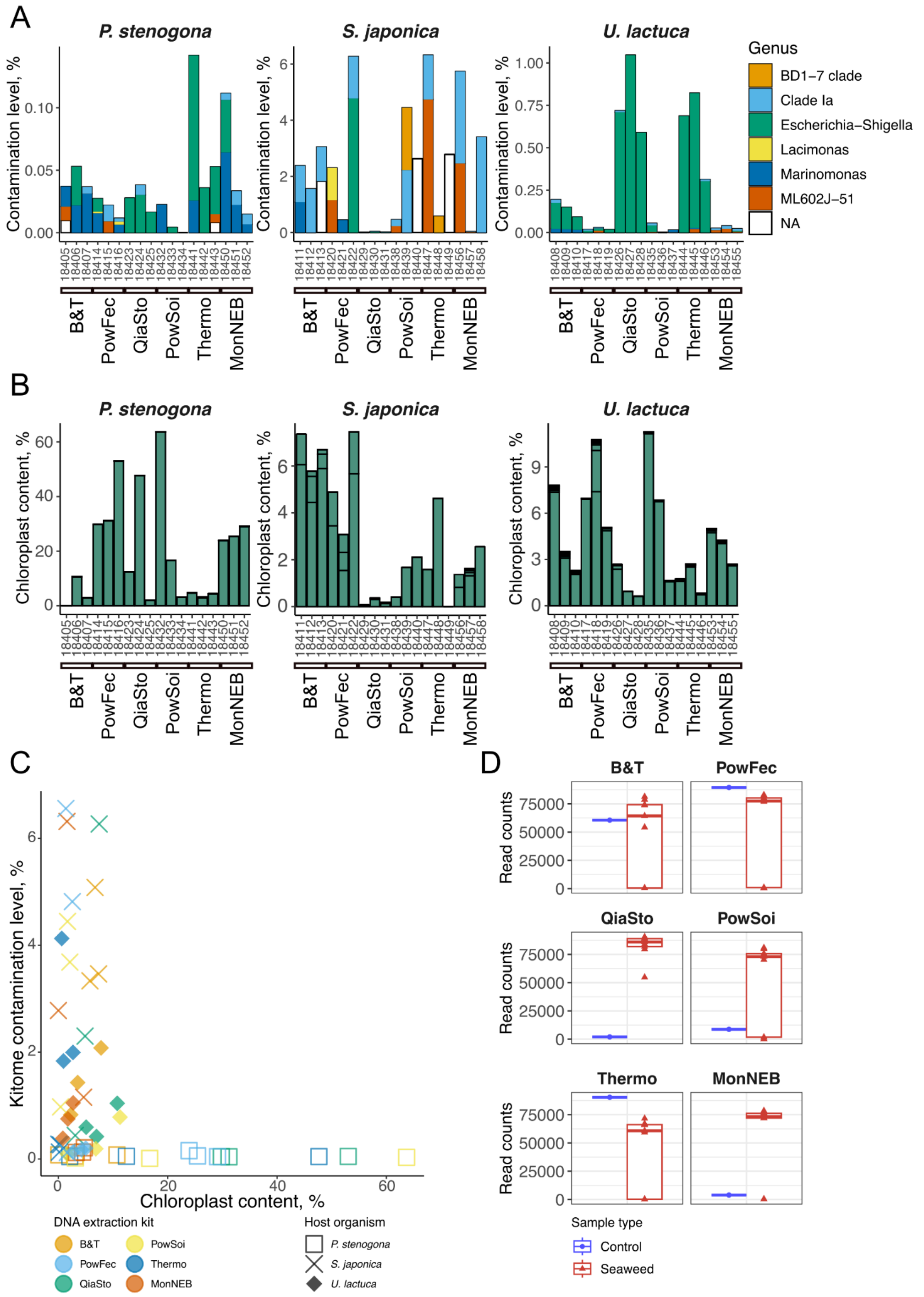

Supplementary Figure 8. Assessment of contamination with “kitome”-derived bacteria and associated chloroplast sequences for different DNA extraction kits tested. (**A**) Contamination levels of macroalgae samples estimated by Decontam. Data is shown for all technical replicates independently. Genera with relative abundances >1% are shown. (**B**) Chloroplast 16S abundance in macroalgae samples. Data is shown for all technical replicates independently. (**C**) Contamination level and chloroplast 16S abundance in macroalgae samples processed with different DNA extraction kits. (**D**) Total read counts after processing raw reads with DADA2 (including denoising, merging, and chimera removal steps) for different DNA extraction kits. Blue and red boxplots represent control and environmental samples, respectively. Individual samples are shown, respectively, with blue dots and red triangles. Data for three technical replicates are shown.

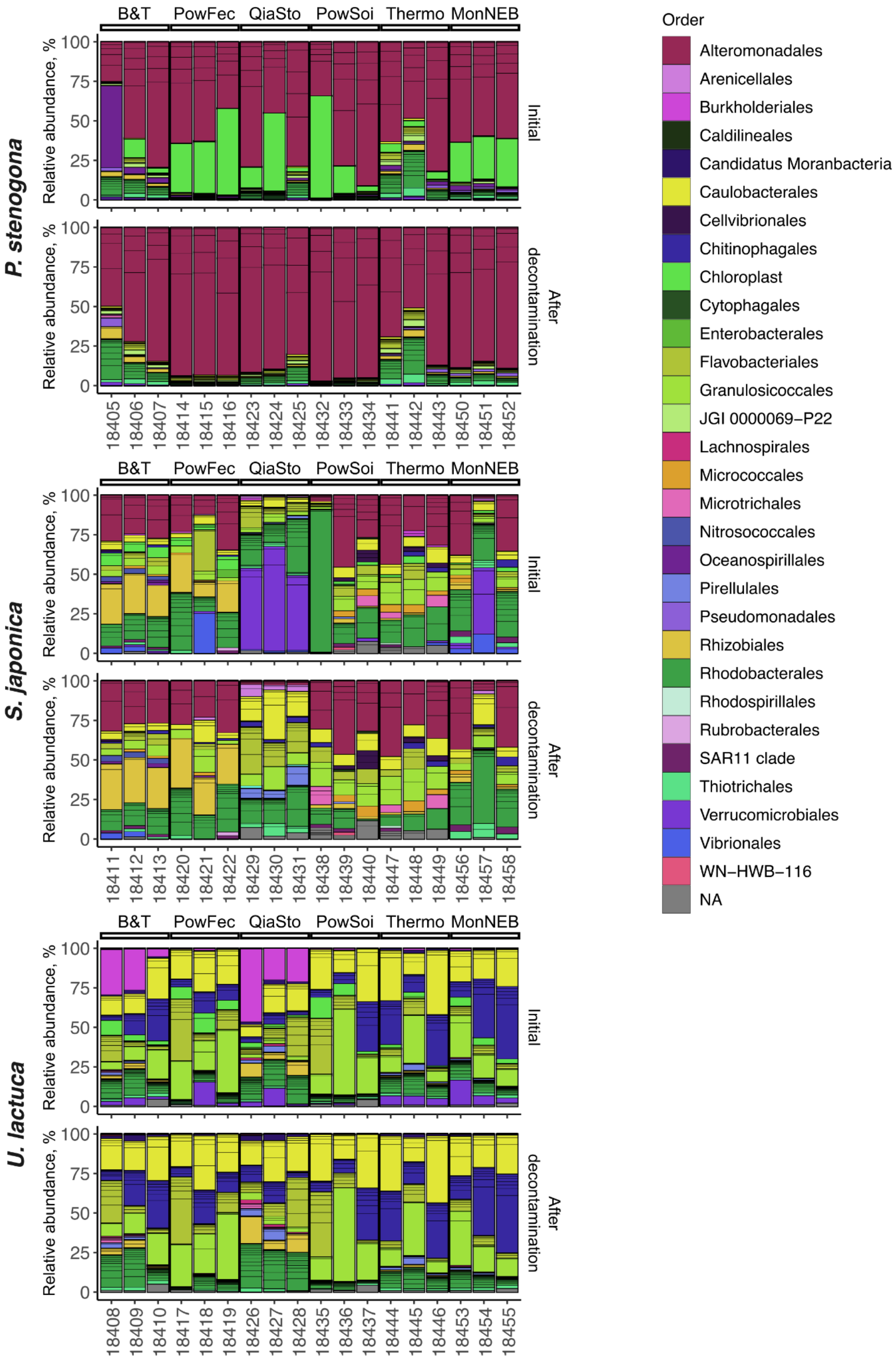

Supplementary Figure 9. Composition of microbial communities associated with macroalgae before (Initial) and after decontamination at order level. Data is shown for all technical replicates independently. Orders with a relative abundance of >1% are shown.

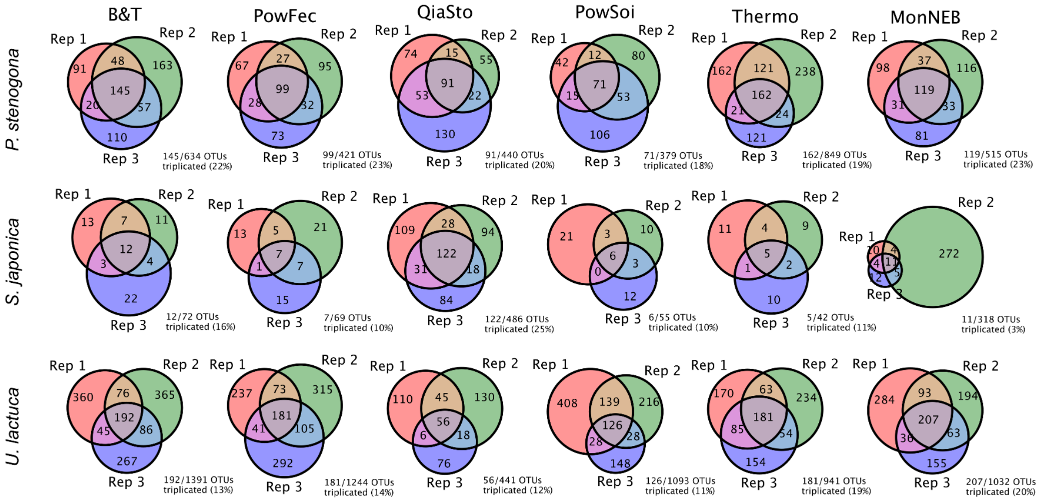

Supplementary Figure 10. Reproducibility of DNA extraction kits. Venn diagrams representing the intersections of lists of non-zero OTUs (OTUs with a non-zero abundance) for three technical replicates obtained with specified DNA-extraction kits. Below each diagram, reproducibility level is shown (in %) as a fraction of non-zero OTUs found in all three replicates from the total number of unique non-zero OTUs found in at least one replicate.

Supplementary Tables

Supplementary Table 1. Performance of different methods for recovery of bacterial cells from macroalgae thalli. All samples were processed with the PowSoi kit (Qiagen).
* - Total amount of purified DNA, using 60 µl of elution buffer.

Macroalgae species	Parameter	Bacterial cells recovery method				
		PBS	PBS + 0.1% BFR	PBS + 1% BFR	Homogenization	Swab
S. japonica	Sample weight, g	1.57± 0.16	1.55± 0.08	1.56± 0.17	1.56± 0.06	1.99± 0.12
	DNA yield, ng*	258.2± 297.1	168.6± 123.7	306.8± 160.1	1024.0± 751.7	591.6± 164.1
	Fragmentation (DIN)	5.5± 0.71	3.65± 3.32	3.37± 2.34	1.03± 0.06	2.97± 2.11
	Quality (260/280)	1.57± 0.43	1.79± 0.37	1.57± 0.12	2.25± 0.51	2.49± 0.22
	Quality (260/230)	0.14± 0.11	0.14± 0.16	0.18± 0.07	0.02± 0.01	0.41± 0.19
U. lactuca	Sample weight, g	5.06± 0.28	5.09± 0.2	5.03± 0.16	5.03± 0.2	5.23± 0.65
	DNA yield, ng*	1158.0± 79.4	1504.0± 174.9	1736.0± 630.6	2806.8± 3652.9	1668.0± 405.7
	Fragmentation (DIN)	3.47± 1.21	3.13± 1.17	4.17± 1.10	4.23± 1.78	4.67± 0.32
	Quality (260/280)	1.87± 0.05	1.77± 0.12	2.09± 0.19	1.60± 0.26	1.61± 0.04
	Quality (260/230)	0.58± 0.39	0.34± 0.07	0.69± 0.57	1.45± 1.07	0.24± 0.12
P. stenogona	Sample weight, g	3.67± 0.16	3.69± 0.15	3.7± 0.14	3.72± 0.11	3.73± 0.1
	DNA yield, ng*	1242.0± 244.4	3216.0± 1514.3	3224.0± 827.6	7788.0± 3174.9	4232.0± 514.2
	Fragmentation (DIN)	1.17± 0.12	1.23± 0.12	1.37± 0.21	2.97± 0.77	1.20± 0.10
	Quality (260/280)	1.91± 0.14	1.95± 0.28	1.86± 0.06	2.01± 0.11	1.88± 0.09
	Quality (260/230)	0.16± 0.10	0.57± 0.54	0.37± 0.31	0.71± 0.55	0.42± 0.40

Supplementary Table 2. Performance of different DNA extraction kits for DNA isolation from bacterial cells recovered from macroalgae thalli. Comparison of DNA yield and quality purified from bacteria associated with three macroalgae species using different DNA extraction kits. NA — No results were obtained due to low DNA quality or the presence of PCR inhibitors.

Macroalgae species	Parameter	DNA extraction kit					
		B&T	PowFec	QiaSto	PowSoi	Thermo	MonNEB
S. japonica	Sample weight, g	2.66± 0.02	2.49± 0.26	2.39± 0.25	2.56± 0.2	2.34± 0.13	2.29± 0.06
	DNA amount, ng	450.8± 299.2	138.7± 100.8	2.9± 3.9	152.4± 18.6	89.2± 24.7	147.9± 103.4
	Quality (260/280)	1.42± 0.32	1.96± 0.82	2.06	1.79± 0.26	1.31± 0.31	1.54± 0.85
	Quality (260/230)	0.46± 0.30	0.04± 0.02	0.04± 0.02	0.21± 0.23	1.52± 1.43	0.09± 0.04
	Fragmentation (DIN)	2.80± 2.78	1.55± 0.78	N/A	1.57± 0.38	4.50± 1.13	2.64± 1.52
U. lactuca	Sample weight, g	1.85± 0.19	1.79± 0.04	1.82± 0.16	1.84± 0.21	1.88± 0.08	1.78± 0.11
	DNA amount, ng	77.5± 42.7	89.9± 82.8	<1	97.6± 56.7	19.1± 3.1	169.2± 93.7
	Quality (260/280)	1.53± 0.30	2.01± 0.50	2.97	1.61± 0.22	0.51	1.77± 0.51
	Quality (260/230)	0.46± 0.38	0.14± 0.10	0.09± 0.02	0.13± 0.18	0.52± 0.04	0.14± 0.16
	Fragmentation (DIN)	1.17± 0.21	1.05± 0.07	N/A	1.30± 0.42	1.50	1.70± 0.40
P. stenogona	Sample weight, g	3.95± 0.03	4.05± 0.05	4.02± 0.07	4.03± 0.04	4.05± 0.03	3.97± 0.09
	DNA amount, ng	3640.0± 1471.7	605.2± 150.6	13.6± 11.4	1146.0± 1596.2	45.7± 14.7	1455.2± 1364.0
	Quality (260/280)	2.35± 0.27	1.81± 0.09	2.96	1.70± 0.05	1.28± 0.52	1.74± 0.06
	Quality (260/230)	1.16± 1.44	0.26± 0.18	0.26± 0.18	0.20± 0.11	0.61± 0.41	1.23± 0.72
	Fragmentation (DIN)	1.90± 0.28	1.53± 0.42	N/A	1.77± 0.60	3.90± 1.61	2.80± 1.06

Supplementary Table 3. Forward (F) and reverse (R) PCR primers, used for PCR amplification, detection of inhibitors and quantitative real-time PCR.

Gene	Primer name	Direction	Sequence 5'- 3'	Source
Bacterial 16S (V3-V4 region)	16S_341_F	F	TCGTCGGCAGCGTCAGATGTGTATAAGAGACAGCCTACGGGNGGCWGCAG	[54]
	16S_785_R	R	GTCTCGTGGGCTCGGAGATGTGTATAAGAGACAGGACTAAACHVGGGTATCTAATCC	[54]

Supplementary Table 4. Read count statistics for negative controls ("kitome") and macroalgae samples. SD — Standard deviation of read counts. M/C — Macroalgae/Control ratio.

Kit	Control read count	Macroalgae mean read count	Macroalgae median read count	Macroalgae SD	M/C Mean ratio	M/C Median ratio
**B&T**	60555	47542	64259	36200	0.8	1.1
**PowFec**	89426	53202	77434	39552	0.6	0.9
**QiaSto**	1993	82385	85942	10968	41.3	43.1
**PowSoi**	8722	50580	73044	37551	5.8	8.4
**Thermo**	90430	43503	60756	32684	0.5	0.7
**MonNEB**	3928	58317	73333	32926	14.8	18.7
